# Loss of RBMS1 promotes anti-tumor immunity through enabling PD-L1 checkpoint blockade in triple-negative breast cancer

**DOI:** 10.1038/s41418-022-01012-0

**Published:** 2022-05-10

**Authors:** Jinrui Zhang, Ge Zhang, Wenjing Zhang, Lu Bai, Luning Wang, Tiantian Li, Li Yan, Yang Xu, Dan Chen, Wenting Gao, Chuanzhou Gao, Chaoqun Chen, Menglin Ren, Yuexia Jiao, Hongqiang Qin, Yu Sun, Lili Zhi, Yangfan Qi, Jinyao Zhao, Quentin Liu, Han Liu, Yang Wang

**Affiliations:** 1 Institute of Cancer Stem Cells and Second Affiliated Hospital, Dalian Medical University, Dalian, 116044 China; 2 Department of Immunology, College of Basic Medical Sciences, Dalian Medical University, Dalian, 116044 China; 3 School of Medicine, Southern University of Science and Technology, Shenzhen, 518035 China; 4 Department of Pathology, First Affiliated Hospital, Dalian Medical University, Dalian, 116044 China; 5 Institute of Genome Engineered Animal Models for Human Diseases, Dalian Medical University, Dalian, 116044 China; 6 CAS Key Laboratory of Separation Science for Analytical Chemistry, Dalian Institute of Chemical Physics, Chinese Academy of Sciences, Dalian, 116023 China

**Keywords:** Cancer, Cell biology

## Abstract

Immunotherapy has been widely utilized in multiple tumors, however, its efficacy in the treatment of triple-negative breast cancers (TNBC) is still being challenged. Meanwhile, functions and mechanisms of RNA binding proteins in regulating immunotherapy for TNBC remain largely elusive. Here we reported that the RNA binding protein RBMS1 is prevalent among immune-cold TNBC. Through a systematic shRNA-mediated screen, we found depletion of RBMS1 significantly reduced the level of programmed death ligand 1 (PD-L1) in TNBC. Clinically, RBMS1 was increased in breast cancer and its level was positively correlated to that of PD-L1. RBMS1 ablation stimulated cytotoxic T cell mediated anti-tumor immunity. Mechanistically, RBMS1 regulated the mRNA stability of B4GALT1, a newly identified glycosyltransferase of PD-L1. Depletion of RBMS1 destabilized the mRNA of B4GALT1, inhibited the glycosylation of PD-L1 and promoted the ubiquitination and subsequent degradation of PD-L1. Importantly, combination of RBMS1 depletion with CTLA4 immune checkpoint blockade or CAR-T treatment enhanced anti-tumor T-cell immunity both in vitro and in vivo. Together, our findings provided a new immunotherapeutic strategy against TNBC by targeting the immunosuppressive RBMS1.

## Introduction

Cancer immunotherapies, including immune checkpoint blockade and CAR-T therapy, have been widely used in multiple cancers [1–4]. However, the immune-cold tumors (e.g. breast cancer) demonstrate a quite poor clinical response to immunotherapies [5]. Although some aggressive triple negative breast cancers (TNBC) are immunogenic, the majority of TNBC patients show limited responses, especially when tumors lack tumor-infiltrating lymphocytes (TILs), failing to stimulate anti-tumor immunity [6]. Therefore, identification of suitable targets that could enhance immunogenicity of those cancer cells, will provide a novel approach to improve anti-tumor immune activities against TNBC.

The TIL response could be regulated by multiple factors that are expressed by cancer cells. The aberrant activation of the inhibitory TIL response is usually known as immune checkpoints, which help cancer cells to evade immune attacks [7]. Programmed death ligand-1 (PD-L1; also known as B7-H1 or CD274) is a 33 kDa type 1 transmembrane protein that binds programmed cell death protein-1 (PD-1) on T cells to create one of the major immune checkpoints, thereby suppressing activation, expansion, and acquisition of effector functions of TILs and enabling cancer cells to evade T cell-mediated immune surveillance [1]. Overexpression of PD-L1 in tumor cells also mediates CAR-T cell exhaustion, resulting in poor therapeutic effects of CAR-T cells in solid tumors [8]. Thus, targeting PD-L1/PD1 axis could reinvigorate exhausted TILs and CAR-T cells in tumor microenvironment, providing promising therapeutic outcomes for cancers [2, 9–11]. However, the response rates from a monotherapy of immune checkpoint blockades are mostly less than 40%, and a large number of patients do not respond well to such therapy [12]. Therefore, investigation of novel immune-checkpoints regulators will offer potential targets for new combinatorial strategies to improve the efficacy of immune checkpoints blockade therapies.

The expression of PD-L1 in cancer cells are regulated by multiple pathways, including genetic, transcriptional and post-transcriptional layers [1, 13]. Recently, post-translational modification of PD-L1 offers further chances to manipulate immune system to eliminate cancer cells [14, 15]. For example, glycogen synthase kinase 3ß (GSK3ß) could directly phosphorylate PD-L1 at the T180 and S184 residues, resulting in PD-L1 polyubiquitination by ß-transducin repeat-containing protein (ß-TRCP) and subsequent degradation [14]. Additionally, ubiquitin aldehyde-binding protein 1 (OTUB1) removes K48-linked ubiquitin chains from PD-L1, thereby suppressing PD-L1 degradation via the ERAD pathway [16]. Accumulating evidence shows that the N-linked-glycosylation of N192, N200, and N219 on PD-L1 enhances its protein stability and interaction with PD-1, leading to cancer immune evasion [14]. Therefore, removal of N-linked-glycosylation of PD-L1 should improve the therapeutic efficacy of immunotherapy. Currently, ß−1,3-N-acetylglucosaminyltransferase 3 (B3GNT3) is one of the reported glycotransferases that mediates PD-L1 glycosylation [14]. Additional targets that regulate PD-L1 glycosylation will provide novel approaches to block the immune checkpoints.

RNA-binding proteins (RBPs) are a diverse group of proteins that participate in regulating gene expression. RBPs interact with RNAs to form ribonucleoprotein complexes to regulate RNA stability, pre-mRNA splicing, RNA editing, translation and so on [17–19]. Strikingly, RBPs play critical roles in tumorigenesis and cancer progression [20–24]. For instance, RBMS1 has been reported to regulate cancer progression through modulating the stability and translation of target mRNAs [25, 26]. Here, we report that RBMS1 ablation enhances the efficacy of anti-CTLA4 and CAR-T immunotherapies by reducing PD-L1 levels through suppressing PD-L1 glycosylation. Hence, our study reveals a novel molecular mechanism for regulating PD-L1 levels by an RNA-binding protein, which has the potential for developing combinatorial cancer immunotherapies against human TNBC by combining RBMS1 depletion with CTLA4 immune checkpoint blockade or CAR-T therapy.

## Results

### Systematic identification of RBMS1 as a key regulator of PD-L1 in cold TNBC

**Fig. 1:**
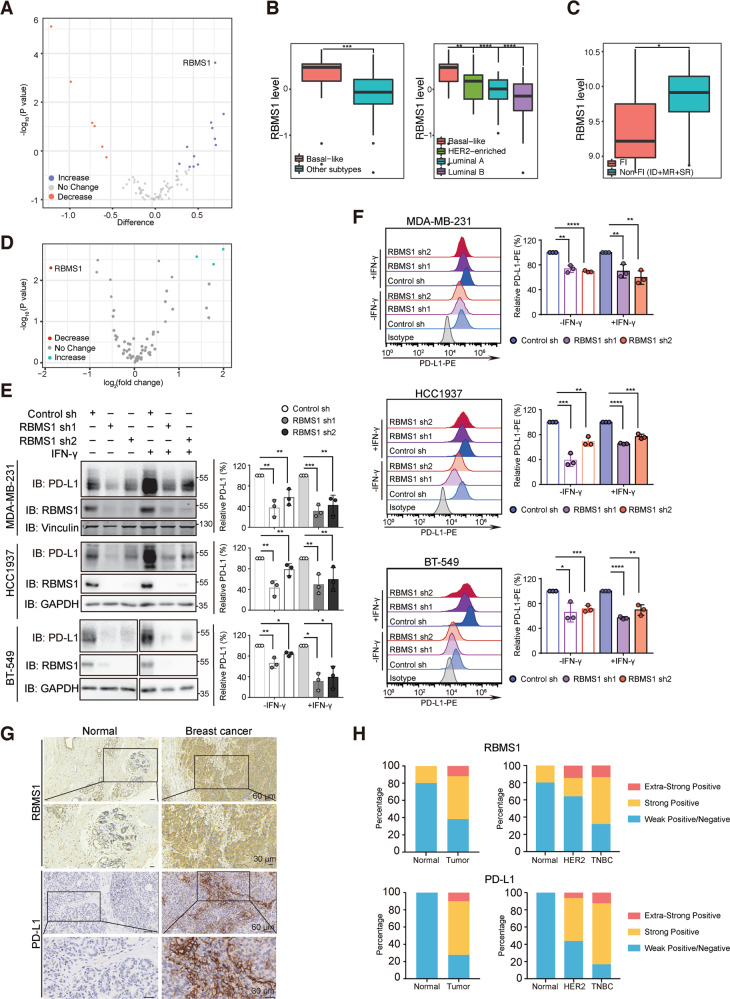
Systematic identification of RBMS1 as a key regulator of PD-L1 in cold TNBC. **A** Volcano plot illustrating the protein levels of 82 RNA binding proteins in basal-like subtype vs luminal A, luminal B, and HER2 subtypes of breast cancer. RBPs levels increased (blue) or decreased (red) are highlighted. **B** The protein level of RBMS1 in basal-like subtype and other subtypes of breast cancer was obtained from (**A**) and plotted (left). The protein level of RBMS1 in basal-like, luminal A, luminal B, or HER2 subtypes of breast cancer was analyzed respectively from (**A**) and plotted (right). **C** The mRNA levels of RBMS1 in FI, and non-FI (SR + MR + ID) were obtained from thirty-eight TNBC patient samples from GSE88847. *P* values were determined using by unpaired *t* test or one-way ANOVA with Dunnett multiple comparisons in (**B**) and (**C**). **D** Volcano plot illustrating the RNA binding proteins whose depletion can regulate the level of PD-L1. RBPs depletion could reduce (red) or increase (blue) the level of PD-L1 are highlighted. **E** The levels of PD-L1 and RBMS1 were measured in RBMS1 stably depleted MDA-MB-231, BT-549, and HCC1937 breast cancer cells in the absence or presence of IFN-γ. Data represent mean ± SD, *n* = 3 independent repeats. *P* values were determined using by One-way ANOVA with Dunnett multiple comparisons. **F** MDA-MB-231, HCC1937, and BT-549 cells with stable depletion of RBMS1 were treated with or without IFN-γ. Cell surface analysis of PD-L1 protein using flow cytometry was shown. Data showed relative fold change in the MFI of PD-L1. Error bar represent mean ± SD. *P* values were determined by One-way ANOVA with Dunnett multiple comparisons. **G** Representative images from immunohistochemical staining of RBMS1 and PD-L1 in breast cancers (*n* = 40) and normal breast tissues (*n* = 10). Scale bars are indicated in the pictures. **H** The quantification of RBMS1 and PD-L1 protein levels in breast cancer and normal breast tissues. The RBMS1 and PD-L1 level were respectively classified into three grades (weak positive/negative, strong positive, extra-strong positive) by results from immunohistochemical staining and plotted. *P < 0.05, **P < 0.01, ***P < 0.001, ****P < 0.0001 for all panels.

Accumulating evidence has demonstrated that a large number of RNA-binding proteins are involved in breast cancer initiation and progression [27–29]. From a bioinformatic analysis of proteomic datasets from eighty-one patients with breast cancer in The Cancer Genome Atlas (TCGA) [30], we found that expression of multiple RNA binding proteins, including RBMS1, DDX6, SND1, and ACO1, was increased in the basal subtype of breast cancer, also known as triple-negative breast cancer (TNBC), as compared to other subtypes (Fig. S1A). Further analysis showed that RBMS1 is the top increased RBP in basal vs other subtypes of breast cancer among the RNA binding proteins analyzed (Fig. 1A, B).

TNBC has been recently subdivided into four subsets according to the tumor immune microenvironment (TIME) in several studies [31, 32], including fully inflamed (FI), stroma restricted (SR), margin-restricted (MR), and immune desert (ID). Both ID and MR are poorly infiltrating tumors with characteristics of fibrosis. The SR subtype is defined based on the infiltration of CD8^+^ T cells in the stroma and low interferon (IFN) signature. However, FI TNBC is the only subtype that has a proinflammatory microenvironment defined according to the type I IFN gene characteristics, CD8^+^ T cell infiltration in the tumor epithelium, satisfactory outcomes [31]. When analyzing the expression of aforementioned RBPs in FI subtype versus non-fully inflamed tumors (ID + MR + SR), we found that in thirty-eight TNBC patient samples from the Gene Expression Omnibus (GEO) database (GSE88847), the mRNA level of RBMS1 was elevated and correlated with the non-fully inflamed phenotype (Figs. 1C and S1B–C), indicating that RBMS1 might play a critical role in regulating immune surveillance in TNBC. These observations suggest that RBMS1 may be involved in suppressing inflammation and anti-tumor immunity in tumor microenvironment.

Consistently, in an independent RNAi screen with an shRNA library to identify RBPs that could regulate the levels of PD-L1 (Fig. S1D), we found that several RBPs could modulate the levels of PD-L1 in MDA-MB-231 TNBC cells, with RBMS1 as one of the top hits whose depletion reduced the level of PD-L1 (Figs. 1D,  S1D and S7). Moreover, in a panel of breast cancer cell lines, the level of RBMS1 was notably elevated in TNBC cells (e.g. MDA-MB-231 and HCC1937) as compared to normal breast cells and other subtype breast cancer cells (Figs. S1E and S7), indicating that RBMS1 might regulate the PD-L1 checkpoint immune surveillance in TNBC.

To further test the role of RBMS1 in regulating PD-L1, we stably knocked down RBMS1 in multiple TNBC cells and investigated the level of PD-L1. As expected, depletion of RBMS1 could significantly decrease the protein level of PD-L1 in MDA-MB-231, HCC1937, and BT-549 cells with or without IFN-γ (Figs. 1E and S7). Similar results were also obtained in mouse 4T1 mammary tumor cells (Figs. S1F and S7). Conversely, overexpression of RBMS1 elevated the level of PD-L1 in multiple breast cancer cells (Figs. S1G–H and S7). We next examined the level of cell surface PD-L1 in RBMS1-depleted MDA-MB-231, HCC1937, BT-549, and 4T1 cells using a flow cytometry assay, and found the level of PD-L1 was also reduced upon the depletion of RBMS1 with or without IFN-γ (Figs. 1F and S1I).

To investigate the potential clinical association between RBMS1 and PD-L1, we measured the protein levels of RBMS1 and PD-L1 on human breast cancer tissue microarrays containing 50 breast cancer patient samples by immunohistochemistry (IHC) assay (Fig. 1G). We showed that >60% of breast cancer samples exhibited strong or extra-strong staining for both of RBMS1 and PD-L1, whereas most of the normal breast tissues (~80%) displayed weak positive/negative staining for RBMS1 and PD-L1 (Fig. 1G, H). Importantly, TNBC patient samples demonstrated much higher levels of RBMS1 and PD-L1 as compared to normal breast tissues and HER2-positive patient samples (Fig. 1H). In addition, our clinical observations demonstrated that RBMS1 is positively correlated to PD-L1 in breast cancer samples (Fig. S1J), suggesting that RBMS1 might be involved in regulating PD-L1 expression in breast cancer, especially TNBC.

### RBMS1 ablation stimulates anti-tumor T cell immunity

**Fig. 2:**
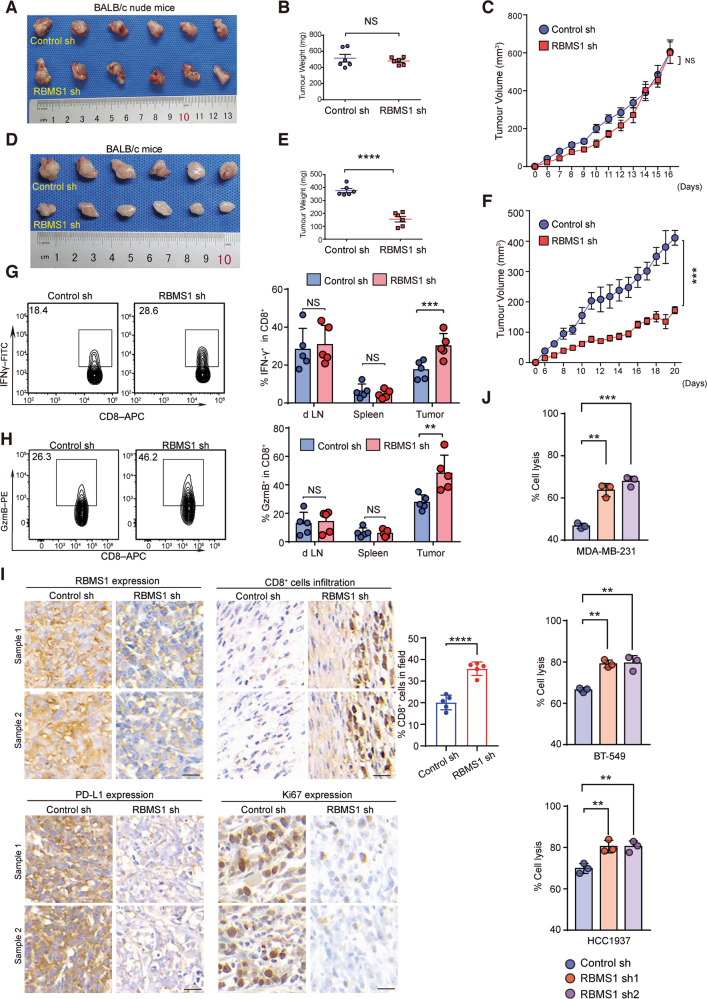
RBMS1 ablation stimulates anti-tumor T cell immunity. **A** Nude mice were subcutaneously injected with 4T1 cells with stable depletion of RBMS1. Pictures of the tumors removed after sixteen days were shown. **B** Tumors were weighed and plotted. **C** The average sizes of tumors were measured every day and plotted (*n* = 6, error bars indicate mean ± SD). **D** BALB/c mice were subcutaneously injected with 4T1 cells with stable depletion of RBMS1. Pictures of the tumors removed after twenty days were shown. **E** Tumors were weighed and plotted. Data represent mean ± SD (*n* = 6). P values were calculated by unpaired *t* test. **F** The average sizes of tumors were measured every day and plotted (*n* = 6, error bars indicate mean ± SD, *P* values were determined by two-way repeated measures ANOVA). **G**–**H** Quantification of intracellular cytokine staining of IFN-γ (**G**) and Granzyme B (**H**) in CD8^+^ T cell populations in the lymph nodes, spleen, and tumor of BALB/c mice. *P* values were calculated by unpaired Student’s *t* test. Error bars denote mean ± SD (*n* = 5). **I** Immunohistochemistry determination of RBMS1, PD-L1, Ki67 expression and CD8^+^ T cell infiltration into tumor tissues. Scale bars, 20 μm. Statistical results indicate means ± SD in each group (*n* = 5). **J** T cell-mediated tumor cell killing assay in MDA-MB-231, BT-549, and HCC1937 cells with stable depletion of RBMS1. Activated Jurkat cells were co-cultured with control or RBMS1-depleted TNBC cells at the T cell to tumor cell ratio of 10:1 (MDA-MB-231) or 20:1 (BT-549 and HCC1937). After 3 days, tumor cells were enumerated by flow cytometry. The quantitative ratio of dead cells is showed by the bar graph. Data represent mean ± SD, *n* = 3 independent repeats. *P* values were determined by One-way ANOVA with Dunnett multiple comparisons. For all panels, *P < 0.05, **P < 0.01, ***P < 0.001, ****P < 0.0001.

To determine whether RBMS1 ablation-mediated PD-L1 downregulation enhances anti-tumor immunity in vivo, we inoculated 4T1 cells with depleted RBMS1 or control into nude mice (immune deficient) (Figs. S2A and S7). We did not observe any significant changes in tumor growth between mice inoculated with RBMS1-depleted or control cells (Fig. 2A–C). Meanwhile, we inoculated RBMS1-depleted or control 4T1 cells into BALB/c mice (immune competent). Interestingly, knockdown of RBMS1 significantly suppressed tumor growth in the immune competent model (Fig. 2D–F), suggesting the differential tumorigenicity was attributed to immune surveillance.

Moreover, we examined the cytotoxic T lymphocytes (CTL) activity by measuring IFN-γ secretion and Granzyme B (GzmB) release. We found that depletion of RBMS1 elevated the IFN-γ secretion and GzmB release in tumors, as compared to control (Fig. 2G, H). The effector function of CD8^+^ T cells in the spleen and lymph nodes were comparable in mice inoculated with RBMS1-depleted or control 4T1 cells (Fig. 2G, H), indicating that the difference in the intratumoral CD8^+^ T cell effector function was due to the tumor microenvironment. To further corroborate our findings, we analyzed tumor tissues by IHC assay and found significant increased infiltration of CD8^+^ T cells in the stroma but decreased expression of PD-L1 and the proliferation marker Ki67 in RBMS1-depleted tumor tissues (Fig. 2I), conforming that knockdown of RBMS1 promoted anti-tumor immunity in the 4T1 tumor model. Consistently, depletion of RBMS1 in MDA-MB-231, BT-549, and HCC1937 cells enhanced the T cell-mediated cancer cell killing in vitro (Figs. 2J, S2B and S7). Similar results were also obtained in 4T1 cells (Fig. S2C). Altogether, loss of RBMS1 stimulates anti-tumor T cell immunity and inhibits tumor growth in an immune-dependent manner.

### RBMS1 regulates PD-L1 protein degradation through modulating its glycosylation

**Fig. 3:**
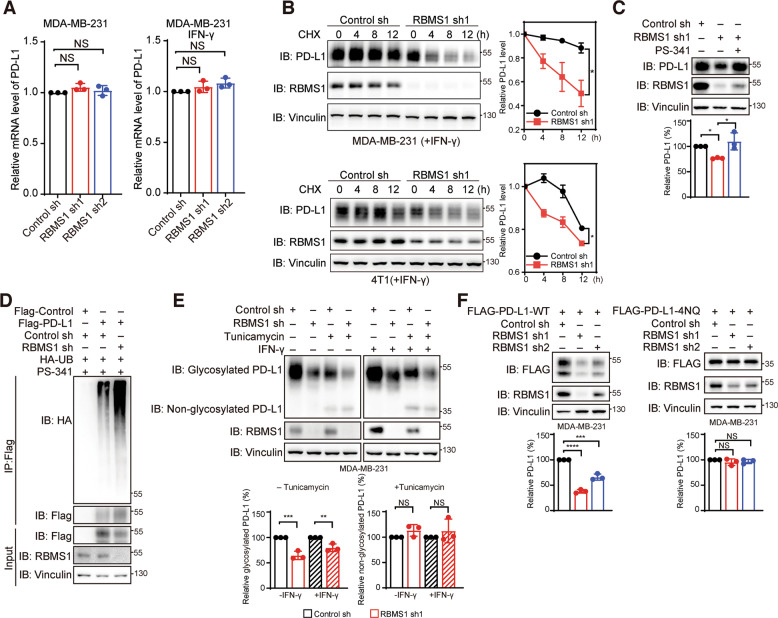
RBMS1 regulates the protein degradation of PD-L1 through modulating its glycosylation. **A** The mRNA levels of PD-L1 in RBMS1-depleted MDA-MB-231 cells with or without IFN-γ treatment were examined using qRT-PCR. Three experiments were conducted with mean ± SD presented. NS denotes non-significant. **B** RBMS1 stably depleted MDA-MB-231 or 4T1 cells were treated with 100 μg/mL cycloheximide (CHX) in the presence of IFN-γ treatment at the indicated time points. PD-L1 and RBMS1 levels were measured by immunoblotting. The intensity of PD-L1 was quantified and plotted. Three experiments were conducted with mean ± SEM presented. *P < 0.05, *P* values were determined by two-way repeated measures ANOVA. **C** The protein level of PD-L1 was measured in RBMS1-depleted MDA-MB-231 cells with or without the treatment of PS-341. **D** Flag-PD-L1 was transiently transfected into RBMS1 stably depleted MDA-MB-231 cells in the presence of PS-341. Then Flag-PD-L1 was immunoprecipitated by anti-FLAG M2-beads followed by immunoblot using antibody against HA-ubiquitin. **E** RBMS1-depleted MDA-MB-231 cells were treated with or without tunicamycin treatment in the absence or presence of IFN-γ. The level of glycosylated or non-glycosylated PD-L1 was measured with a western blot assay. **F** RBMS1-downregulated MDA-MB-231 cells were transiently transfected with wild-type PD-L1 or 4NQ-mutant-PD-L1 vector. The level of wild-type PD-L1 or 4NQ-mutant-PD-L1 was examined with a western blot approach. For panels (**C**, **E**, **F**), data represent mean ± SD, *n* = 3 independent repeats. *P* values were determined using by One-way ANOVA with Dunnett multiple comparisons, *P < 0.05, **P < 0.01, ***P < 0.001, ****P < 0.0001.

Since depleted RBMS1 significantly reduced the level of PD-L1, we sought to determine how RBMS1 affects PD-L1 level. We examined the mRNA levels of PD-L1 in multiple TNBC cells with stable knockdown of RBMS1. Interestingly, the mRNA level of PD-L1 was not influenced by reduced RBMS1 in MDA-MB-231, BT-549, and HCC1937 TNBC cells, as well as mouse 4T1 cells, in the absence or presence of IFN-γ (Figs. 3A and S3A). Conversely, overexpression of RBMS1 could not affect the mRNA level of PD-L1 either (Fig. S3B).

We investigated the protein stability of PD-L1 upon RBMS1 depletion. In the presence of protein synthesis inhibitor cycloheximide, the turnover rate of PD-L1 was much faster in MDA-MB-231 and 4T1 cells with RBMS1 downregulation as compared to control cells in the absence or presence of IFN-γ (Figs. 3B,  S3C and S7). We further examined whether the RBMS1-mediated degradation of PD-L1 is through ubiquitin pathway. As expected, RBMS1 depletion-induced downregulation of PD-L1 could be prevented by the treatment of PS-341, a proteosome inhibitor (Fig. 3C and S7). Additionally, depletion of RBMS1 promoted PD-L1 ubiquitination as compared to control (Figs. 3D, S3D and S7). Taken together, our data suggest that loss of RBMS1 enhances PD-L1 protein degradation through the proteosome pathway.

Glycosylation has been previously reported to play important roles in regulating PD-L1 protein stability [14]. We thus sought to investigate whether RBMS1 affects PD-L1 stability via modulating its glycosylation. We treated cells with tunicamycin, a glycosylation inhibitor, and found that the protein bands corresponding to PD-L1 shifted down to a smaller size similar to that of the non-glycosylated form (Figs. 3E and S7). Strikingly, RBMS1 was not capable of affecting the level of non-glycosylated PD-L1 upon the tunicamycin treatment with or without IFN-γ (Figs. 3E and S7). We further validated this observation by examining the level of exogenously expressed wild type or non-glycosylated form (4NQ) PD-L1 [14], and found that RBMS1 only affected the protein level of glycosylated wild type PD-L1, but not PD-L1 with 4NQ mutation that cannot be glycosylated in both MDA-MB-231 and HEK293T cells (Figs. 3F, S3E and S7). Altogether, our results imply that RBMS1 regulates PD-L1 protein degradation through modulating its glycosylation.

### RBMS1 controls the mRNA stability of B4GALT1 glycosyltransferase to mediate PD-L1 glycosylation

**Fig. 4:**
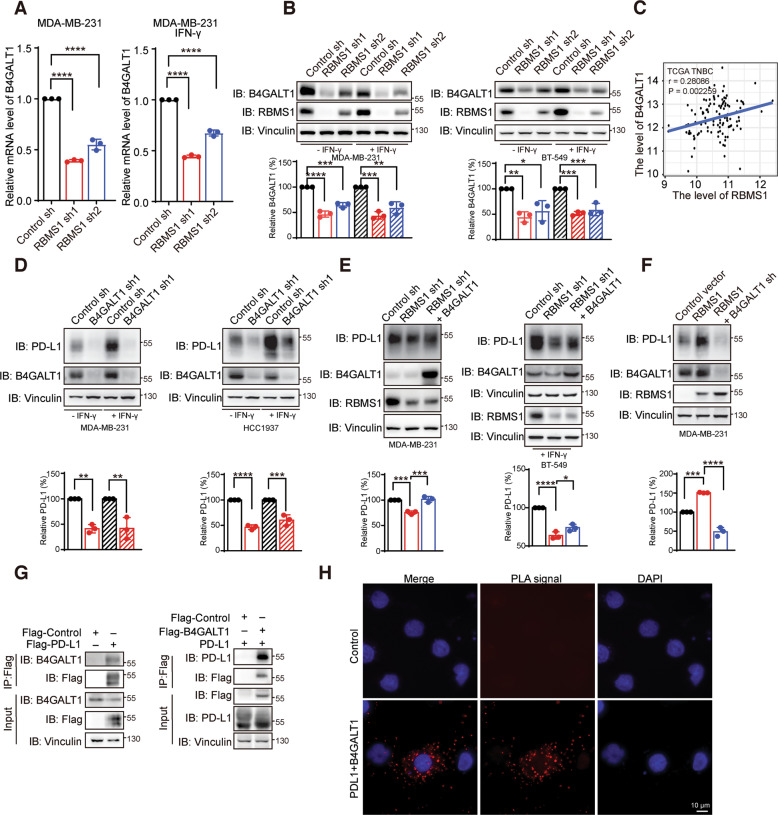
RBMS1 regulates PD-L1 glycosylation through the glycosyltransferase B4GALT1. **A** The level of B4GALT1 was examined in RBMS1-depleted MDA-MB-231 cells in the presence or absence of IFN-γ treatment. Data represent mean ± SD, *n* = 3 independent repeats. *P* values were determined by One-way ANOVA with Dunnett multiple comparisons. *P < 0.05, **P < 0.01, ***P < 0.001, ****P < 0.0001. **B** The protein levels of B4GALT1 and RBMS1 were measured in RBMS1 stably depleted MDA-MB-231 and BT-549 cells with or without treatment of IFN-γ. **C** Correlation of B4GALT1 and RBMS1 levels were analyzed using TCGA TNBC dataset. **D** The protein levels of PD-L1 and B4GALT1 were determined in B4GALT1 stably depleted MDA-MB-231 and HCC1937 cells with or without treatment of IFN-γ. **E** The protein levels of PD-L1, B4GALT1, and RBMS1 were examined in RBMS1 stably depleted MDA-MB-231 and BT-549 cells with or without re-expression of B4GALT1. **F** The protein levels of PD-L1, B4GALT1, and RBMS1 were measured in RBMS1 stably overexpressed MDA-MB-231 cells with or without downregulation of B4GALT1. **G** Immunoprecipitation was performed in HEK293T cells expressing Flag-PD-L1 (left), or Flag-B4GALT1 and PD-L1 (right), and the precipitated-complexes were analyzed. **H** PLA was performed in HCC1937 cells to examine the interaction between PD-L1 and B4GALT1. PLA signals were shown in red and the nuclei in blue. For panels (**B**, **D**, **E**, **F**), data represent mean ± SD, *n* = 3 independent repeats. *P* values were determined using by One-way ANOVA with Dunnett multiple comparisons, *P < 0.05, **P < 0.01, ***P < 0.001, ****P < 0.0001.

To mechanistically study how RBMS1 regulates PD-L1 glycosylation, we performed an RNA-seq assay with RBMS1-depleted or control MDA-MB-231 cells. We found that the glycosyltransferase beta-1,4-galactosyltransferase 1 (B4GALT1) was one of the top hits whose expression level was significantly decreased upon RBMS1 depletion in breast cancer cells (Figs. 4A and S4A–B). However, levels of other members of the B4GALT1 family (e.g. B4GALT3, B4GALT4, and B4GALT7) and B3GNT3, the known glycosyltransferase of PD-L1, were not affected by RBMS1 (Fig. S4C), indicating that RBMS1 specifically regulates the level of B4GALT1. In addition, the protein level of B4GALT1 was reduced accordingly in the absence or presence of IFN-γ (Figs. 4B and S7). Conversely, overexpression of RBMS1 increased the expression level of B4GALT1 in TNBC cells (Figs. S4D and S7). We subsequently analyzed the clinical correlation of RBMS1 and B4GALT1 in TCGA breast cancer dataset, and found that the expression level of B4GALT1 is positively correlated to the level of RBMS1, especially in TNBC dataset (Fig. 4C). Knockdown of B4GALT1 could indeed reduce PD-L1 glycosylation in multiple breast cancer cells in the absence or presence of IFN-γ (Figs. 4D, S4E and S7), whereas overexpression of B4GALT1 elevated the glycosylation of PD-L1 (Figs. S4F and S7). We further re-expressed B4GALT1 in RBMS1-depleted breast cancer cells, and found that re-expression of B4GALT1 rescued RBMS1 depletion-induced decrease of PD-L1 glycosylation (Figs. 4E and S7). Meanwhile, depletion of B4GALT1 in breast cancer cells with overexpression of RBMS1 notably reduced the glycosylation of PD-L1 (Figs. 4F, S4G and S7). Additionally, PD-L1 interacted with B4GALT1 in an immunoprecipitation assay (Fig. 4G and S7). Such interaction was also observed in HCC1937 and MDA-MB-231 cells in a Proximity Ligation Assay (PLA), a powerful tool that allows in situ detection of endogenous protein interactions with high specificity and sensitivity (Figs. 4H and S4H). Taken together, our data suggest that B4GALT1 might be a novel glycosyltransferase that regulates PD-L1 glycosylation, which could be controlled by RBMS1.

**Fig. 5:**
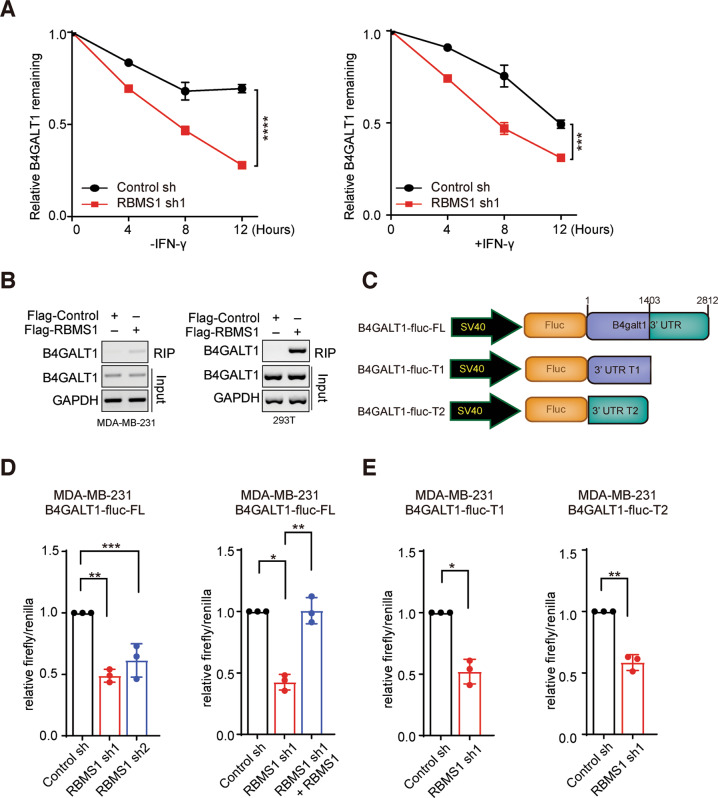
RBMS1 controls the mRNA stability of B4GALT1. **A** RBMS1-depleted MDA-MB-231 cells were treated with actinomycin D as indicated times in the absence (left) of presence (right) of IFN-γ treatment. The mRNA expression levels of B4GALT1 were examined using qRT-PCR. Error bars are mean ± SD from three biologically independent samples. *P* values were determined using two-way repeated measures ANOVA. **B** Binding of *B4GALT1* 3′-UTR with RBMS1 is examined by RNA-immunoprecipitation in MDA-MB-231 and HEK293T cells expressing FLAG-RBMS1. **C** Schematic of B4GALT1 luciferase reporter plasmids. B4GALT1-fluc-FL (the full length of 3′-UTR); B4GALT1-fluc-T1 (1st−1403th nt region of 3′-UTR); B4GALT1-fluc-T2 (1404th-2812th nt region of 3′-UTR). **D** The luciferase reporter B4GALT1-fluc-FL was transiently transfected into RBMS1 stably depleted MDA-MB-231 cells with or without re-expression of RBMS1. The relative luciferase activities were determined by calculating the ratio of firefly luciferase activities over renilla luciferase activities. Error bars are mean ± SD from three biologically independent samples. *P* values were determined by One-way ANOVA with Dunnett multiple comparisons. **E** The luciferase reporters B4GALT1-fluc-T1 and B4GALT1-fluc-T2 were transiently transfected into RBMS1 stably depleted MDA-MB-231 cells. The relative luciferase activities were determined by calculating the ratio of firefly luciferase activities over renilla luciferase activities. Three independent experiments were conducted, with the mean ± SD of relative luciferase activities were shown. P values from a two-sided unpaired *t* test. For panels (**A**, **D**, **E**) *P < 0.05, **P < 0.01, ***P < 0.001, ****P < 0.0001.

We tested whether RBMS1 modulates the mRNA levels of B4GALT1 through regulating the stability of B4GALT1 mRNA. We treated RBMS1-depleted or control cells with actinomycin D, an RNA synthesis inhibitor, and found that RBMS1 ablation significantly promoted the degradation of B4GALT1 mRNA in the absence or presence of IFN-γ (Figs. 5A and S5A). We further carried out RNA-immunoprecipitation (RIP) assay, revealing that RBMS1 could indeed interact with the 3′-UTR of B4GALT1 in multiple cells (Figs. 5B, S5B and S7). Subsequently, we generated luciferase reporters that contained the full-length or truncated 3′-UTR of B4GALT1 respectively (Fig. 5C). We conducted luciferase reporter assay and found that the activity of luciferase reporter B4GALT1-fluc-FL was significantly inhibited by depleted RBMS1 in multiple cells (Figs. 5D, S5C–D and S7), which could be rescued with re-expression of RBMS1 (Figs. 5D, S5C and S7). Meanwhile, since both of the truncated 3′-UTR portions (T1 and T2) included multiple RBMS1 binding sites, the activity of luciferase reporters B4GALT1-fluc-T1 and B4GALT1-fluc-T2 was both significantly suppressed in RBMS1 depleted cells (Figs. 5E, S5E and S7), suggesting that RBMS1 regulates the stability of B4GALT1 mRNA through its 3′-UTR.

### Loss of RBMS1-stimulated anti-tumor T cell immunity is reversed by re-expression of B4GALT1

**Fig. 6:**
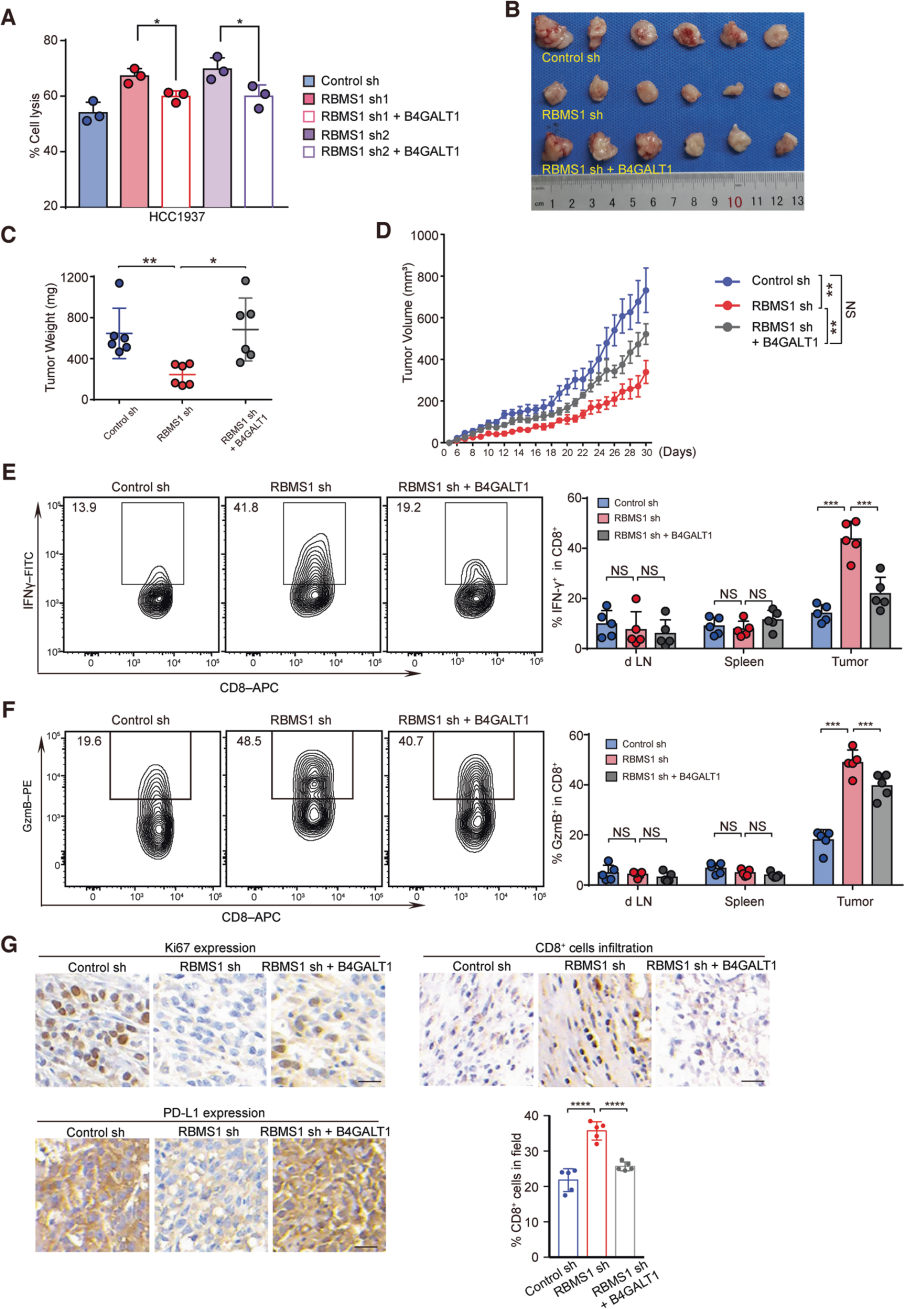
Loss of RBMS1-stimulated anti-tumor T cell immunity is reversed by re-expression of B4GALT1. **A** T cell-mediated tumor cell killing assay in HCC1937 cells with stable depletion of RBMS1 with or without re-expression of B4GALT1. The quantitative ratio of dead cells is showed by the bar graph. Data represent mean ± SD, *n* = 3 independent repeats. *P* values were determined by One-way ANOVA with Tukey’s multiple comparisons. **B** BALB/c mice were subcutaneously injected with RBMS1-depleted 4T1 cells with or without re-expression of B4GALT1. Pictures of the tumors removed after thirty days were shown. **C** Tumors were weighed and plotted. Data represent mean ± SD (*n* = 6), *P* values were determined by One-way ANOVA with Tukey’s multiple comparisons. **D** The average sizes of tumors were measured every day and plotted (*n* = 6, error bars indicate mean ± SD, *P* values were determined by two-way repeated measures ANOVA. **E**–**F** Quantification of intracellular cytokine staining of IFN-γ (**E**) and Granzyme B (**F**) in CD8^+^ T cell populations in the lymph nodes, spleen, and tumor of BALB/c mice. Error bars denote mean ± SD (*n* = 5). **G** Immunohistochemistry determination of PD-L1, Ki67 expression and CD8^+^ T cell infiltration into tumor tissues. Scale bars, 20 μm. Statistical results indicate means ± SD in each group (*n* = 5). Statistically significant by One-way ANOVA with Tukey’s multiple comparisons. For all panels, *P < 0.05, **P < 0.01, ***P < 0.001, ****P < 0.0001.

To determine whether B4GALT1 participates in RBMS1-mediated anti-tumor T-cell immunity, we first re-expressed B4GALT1 in RBMS1-depleted breast cancer cells, and found that overexpression of B4GALT1 significantly reduced the T-cell-mediated cancer cell killing in vitro in HCC1937 and MDA-MB-231 cells (Figs. 6A and S6A). Similar results were also observed in RBMS1-depleted 4T1 cells with re-expression of B4GALT1 (Fig. S6B). We further inoculated RBMS1-depleted 4T1 cells with or without re-expressed B4GALT1 into BALB/c mice. As expected, re-expression of B4GALT1 promoted RBMS1-depleted 4T1 tumor growth in the immune competent model (Figs. 6B–D). Importantly, we determined CTL activity by measuring IFN-γ and GzmB release and showed that re-expression of B4GALT1 decreased IFN-γ and GzmB release, as compared to control (Figs. 6E, F). Moreover, we analyzed tumor tissues by an IHC assay and found that RBMS1-depletion induced reduction of PD-L1 and elevated infiltration of CD8^+^ T cells could be reversed by re-expression of B4GALT1, resulting in increased levels of PD-L1 and Ki67, but decreased infiltration of CD8^+^ T cells (Fig. 6G). Collectively, RBMS1 ablation reduces the level of B4GALT1 to inhibit the glycosylation of PD-L1, thereby stimulating the anti-tumor T-cell immunity.

### Depletion of RBMS1 combined with CTLA4 blockade effectively suppresses tumor growth in syngeneic mouse model

**Fig. 7:**
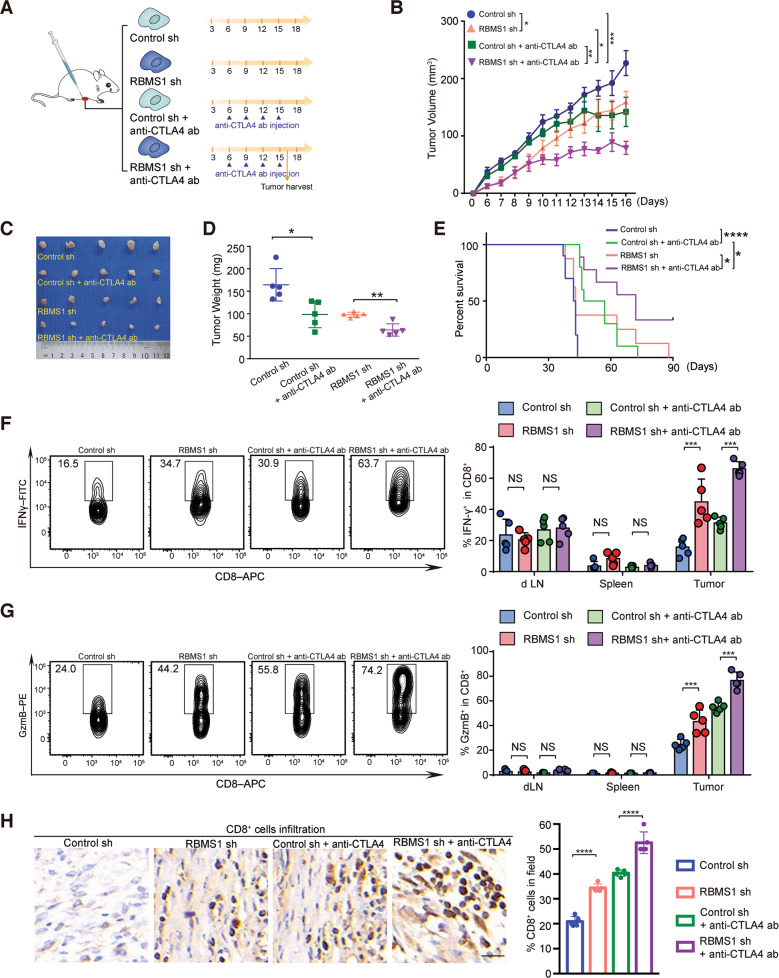
Inhibition of RBMS1 sensitizes the CTLA4 blockade and CAR-T therapy efficacy. **A** The schematic of the xenograft mouse model and dosage regimen. Mice bearing control or RBMS1-depleted 4T1 tumors were treated with anti-CTLA4 antibody. **B** The tumor growth of 4T1 cells with stable depletion of RBMS1 in BALB/c mice following treatment with or without anti-CTLA-4 antibody. The average sizes of xenograft tumors were measured every day and plotted (*n* = 5, error bars indicate mean ± SD, *P* values were determined by two-way repeated measures ANOVA. **C** Pictures of tumors removed after sixteen days were shown. **D** Tumors were weighed and plotted. Data represent mean ± SD (*n* = 5), *P* values were determined by One-way ANOVA with Tukey’s multiple comparisons. **E** Survival of mice bearing syngeneic RBMS1-depleted 4T1 tumor following treatment with or without anti-CTLA4 antibody. Significance was determined by log-rank test. *P < 0.05; *n* = 10 mice per group. **F**, **G** Quantification of intracellular cytokine staining of IFN-γ (**F**) and Granzyme B (**G**) in CD8^+^ T cell populations in the lymph nodes, spleen, and tumor of BALB/c mice. *P < 0.05, Error bars denote mean ± SD (*n* = 5). *P* values were calculated by One-way ANOVA with Tukey’s multiple comparisons. **H** Immunohistochemistry determination of CD8^+^ T cell infiltration into tumor tissues. Scale bars, 20 μm. Statistical results indicate means ± SD in each group (*n* = 5). For all panels, *P < 0.05, **P < 0.01, ***P < 0.001, ****P < 0.0001.

Immune inhibitory checkpoints PD-L1 and CTLA4 function through distinct mechanisms. Recently, combined blockade of PD-L1 and CTLA4 have been demonstrated to significantly improve immunotherapy efficacy [33, 34]. Since we uncovered that depleted RBMS1 reduces PD-L1 level, thereby promoting anti-tumor T-cell immunity, we chose CTLA4 blockade for the combination therapy with RBMS1 depletion in the mouse 4T1 engraftment model. BALB/c mice were inoculated with RBMS1-depleted or control 4T1 cells. When tumors were palpable, mice were treated with control or anti-CTLA4 antibody as indicated (Fig. 7A). Depletion of RBMS1 reduced tumor size and inhibited 4T1 cell-induced tumor growth (Figs. 7B–D). Such downregulated RBMS1-induced efficacy was similar to that of the anti-CTLA4 antibody treatment. Strikingly, the application of RBMS1-depletion with CTLA4 blockade demonstrated a significant improvement in tumor burden and survival rate of mice (Fig. 7B–E). Consistent with our mechanistic findings, flow cytometry analysis of mice tumor tissues revealed that depleted RBMS1 elevated CTL activities (Fig. 7F, G). As for the T-cell activation, the effects of RBMS1 depletion were similar to or better than that of CTLA4 blockade as judged by the levels of IFN-γ and GzmB (Fig. 7F, G). To further validate the combinatorial effect of RBMS1 depletion and CTLA4 blockade, we analyzed tumor tissues by an IHC assay and found the combination therapy with RBMS1-depletion and anti-CTLA4 antibody significantly promoted the infiltration of CD8^+^ T cells as compared to the application of CTLA4 blockade only (Fig. 7H). RBMS1 ablation combined with CTLA4 blockade significantly promoted the CTL activity (Fig. 7B–H). Taken together, our data suggest that RBMS1 depletion has the potential to promote the efficacy of anti-CTLA4 antibody therapy.

### RBMS1 depletion enhances anti-tumor activity of B7-H3.CAR-T cells targeting human TNBC

**Fig. 8:**
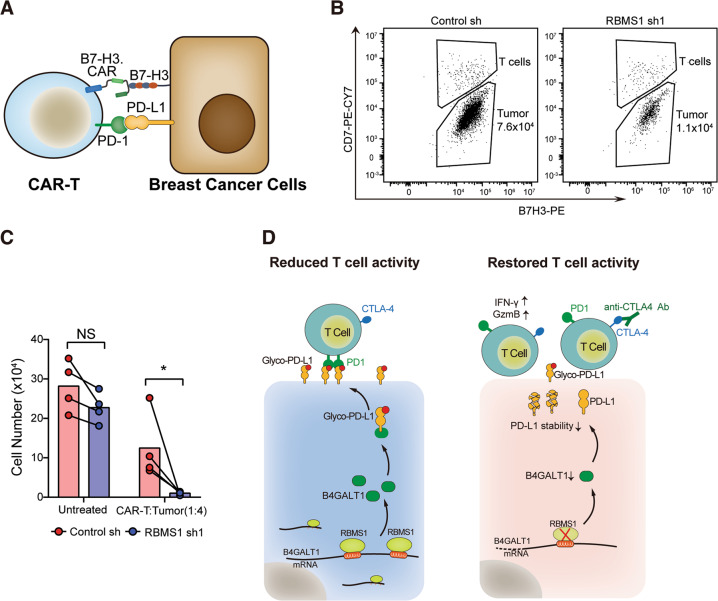
RBMS1 depletion enhances anti-tumor activity of B7-H3.CAR-T cells targeting human TNBC. **A** Schematic of CAR-T therapy in breast cancer. **B**, **C** MDA-MB-231 cells with stable depletion of RBMS1 or control co-cultured with B7H3-specific CAR-T cells (*n* = 4). CD7^+^ CD276dim cells were gated as CAR-T cells while CD7^−^ CD276hi cells were gated as tumor cells. Representative flow cytometry plot (**B**) and quantification of residual tumor cells (**C**) are illustrated. Numbers in panel I indicate absolute count of tumor cells. As control, we used tumor cells without T cells (Untreated). *P < 0.05. **D** Schematic of how RBMS1 regulates the level of PD-L1 through B4GALT1 and modulates anti-tumor T cell immunity in TNBC.

CAR-T cell therapy is effective against hematologic malignancies but its clinical efficacy in solid tumors is limited. This might be attributed to CAR-T cells exhaustion characterized by upregulation of inhibitory receptors (e.g. PD-1) and loss of effector functions. Several preclinical studies suggest that PD-1/PD-L1 blockade may synergize with CAR-T cell therapy to improve its anti-tumor activity [10, 11]. To investigate whether RBMS1-depletion in tumor cells could enhance CAR-T cell activity, B7-H3 specific CAR-T cells (B7H3.CAR-T cells) [35] were co-cultured with RBMS1-depleted or control MB-MDA-231 cells expressing B7-H3 antigen (Fig. 8A). Importantly, depletion of RBMS1 significantly augmented the anti-tumor effect of B7-H3.CAR-T cells at relatively low T-cell to tumor cell ratio (1:4) (Fig. 8B–C). These data suggest this combinatorial strategy have significant clinical potential in improving CAR-T therapy in TNBC.

## Discussion

Immune checkpoints (e.g. PD-L1/PD-1) are regulated by multiple factors, helping cancer cells evade immune attacks. Blockade of immune checkpoints could provide promising therapeutic outcomes for multiple cancers [36–39]. However, few studies have demonstrated that RBPs play critical roles in immune checkpoints regulation. On the basis of unbiased bioinformatic analyses of RBPs in breast cancers with distinct immune status, we identified RBMS1 to be accumulated in TNBC tumors. Using an shRNA library of RBPs, we systematically revealed that RBMS1 could modulate the level of PD-L1 in TNBC cells. Specifically, loss of RBMS1 significantly decreases PD-L1 protein level, thereby enhancing the efficacy of anti-CTLA4 immunotherapy in TNBC. We showed that RBMS1 binds to the 3′-UTR of the glycosyltransferase B4GALT1, which in turn stabilizes its mRNA. Subsequently, the elevated B4GALT1 promotes PD-L1 glycosylation and increases PD-L1 protein stability, preventing cancer cells from immune attacks (Fig. 8D). However, when RBMS1 is depleted, it destabilizes B4GALT1 transcript, resulting in the suppression of glycosylation and increased degradation of PD-L1, thus promoting anti-tumor T-cell immunity and sensitizing TNBC cells to anti-CTLA4 therapy (Fig. 8D). Altogether, our data establish RBMS1 as a novel immune checkpoint regulator with therapeutic potentials and clinical values for TNBC.

RBMS1 has been previously reported to inhibit colon cancer metastasis with clinical utility for risk stratification of patients [25]. Meanwhile, our recent study revealed that RBMS1 regulates lung cancer ferroptosis through translational control of SLC7A11 [26]. However, the expression and function of RBMS1 in breast cancer is less understood. We observed that RBMS1 is highly expressed in breast cancer samples, especially in TNBC samples, which is positively correlated to PD-L1 level. RBMS1 ablation significantly reduced PD-L1 level, thus to stimulate CD8^+^ CTL population and Granzyme B release and enhance the T-cell-mediated cancer cell killing, suggesting that RBMS1 plays an important role in breast cancer immunotherapy regulation.

PD-L1 could be regulated by glycosylation, which inhibits 26 S proteasome-mediated PD-L1 protein degradation, leading to T-cell immunosuppression of cancer cells. B3GNT3 was identified as one of the glycosyltransferases of PD-L1, whereas B3GNT3-downregulation enhanced cytotoxic T-cell-mediated anti-tumor immunity. Our studies discovered B4GALT1 as a novel glycosyltransferase of PD-L1. Depletion of RBMS1 significantly destabilized B4GALT1 mRNA, and reduced PD-L1 glycosylation, promoting the ubiquitination and degradation of PD-L1 in TNBC.

In addition to the underappreciated RBMS1, we also identified some other RNA binding proteins, which could regulate PD-L1 level, including RBFOX2, RPS10, RPS5. Previously, some of the identified RBPs have been shown to participate in cancer progression. For example, RBFOX2 promotes nasopharyngeal carcinogenesis through regulating alternative splicing [40]. However, it is still largely unknown whether targeting RBPs could promote tumor-infiltrating cytotoxic T-cell immune response. Thus, further investigation of RBPs in regulating immune surveillance might provide novel targets to improve the efficacy of cancer immunotherapy.

## Materials and methods

### Reagents

Tunicamycin, Cycloheximide and Actinomycin D were obtained from APExBio (B7414, A8244 and A4448). Recombinant human interferon γ and recombinant mouse interferon γ were obtained from Meilun Biotechnology co., Ltd. Dalian (MB5954 and MA0620). Bortezomib (PS-341) was obtained from Selleck (S1013).

### Cell culture

Cell lines used in this study were obtained from the American Type Culture Collection (ATCC) and cultured under standard culture conditions in culture medium recommended by the ATCC. MDA-MB-231 was cultured with Leibovitz’s L-15 medium (GIBCO) supplemented with 10% FBS (BI) at 37 °C without CO_2_. HCC1937 was cultured with RPMI-1640 media (GIBCO) supplemented with 10% FBS, while BT-549 was maintained using RPMI-1640 media (GIBCO) supplemented with 0.023 U/ml insulin and 10% FBS. 4T1 and HEK293T cell lines were cultured with DMEM (GIBCO) media supplemented with 10% FBS. All cells except MDA-MB-231 were incubated in a humidified atmosphere containing 5% CO_2_ at 37 °C.

### Plasmid constructions and generation of stable cell lines

For RBMS1 (or B4GALT1) knockdown, shRNA targeting RBMS1 (or B4galt1) were cloned into pLKO.1 plasmid. MDA-MB-231, BT-549, HCC1937 and HEK293T were infected by lentiviral shRNAs to generate stable cell lines with RBMS1 (or B4GALT1) knockdown. In brief, target-specific shRNA vectors (pLKO.1) were used to transfect HEK293T cells together with lentivirus packaging plasmids according to the manufacturer’s instructions. Collected viruses were used to infect MDA-MB-231, BT-549, HCC1937 or HEK293T cells and positive cells were selected with puromycin incubation at 2 μg/ml. pLKO.1 empty vector was included as negative control. Stable cell lines were routinely maintained in culture media supplemented with 1 μg/ml of puromycin throughout all experiments. To generate the mammalian expression plasmid pCDH-Flag-RBMS1 and pCDH-Flag-B4GALT1, RBMS1 and B4galt1 cDNA were PCR amplified and then cloned into the lentivirus vector pCDH-CMV-MCS-EF1-Puro with N-terminal Flag tag with restriction enzymes *Nhe* I and *Not* I. MDA-MB-231, BT-549 or HCC1937 were infected by lentiviral pCDH-Flag-RBMS1(or pCDH-Flag-B4GALT1) to generate stable cell lines with RBMS1(or B4GALT1) overexpression.

To generate pCDH-PD-L1 or pCDH-PD-L1-3×FLAG plasmid, PD-L1 were amplified and cloned into pCDH-CMV-MCS-EF1-Puro or pCDH-CMV-MCS-EF1-Puro with C-terminal 3×Flag tag.

To generate B4GALT1 luciferase reporter, the DNA fragment of human B4galt1 gene 3′-UTR full length and truncates (T1 and T2) were amplified by PCR and cloned into the *Xba* I sites of the pGL3-Control plasmid. All constructs were confirmed by DNA sequencing. Primers for PCR amplification, and shRNAs are listed in Supplementary Table 1.

### Western blot

Western blotting was performed using standard methods. The following antibodies were used: RBMS1 (Abcam ab150353), PD-L1(CST 13684), PD-L1 (Abcam ab213480), B4GALT1 (Santa Cruz sc-515551), VINCULIN (Proteintech 66305-1-Ig), GAPDH (Proteintech 60004-1-Ig), TUBULIN (ABclonal AC006). Secondary antibodies were conjugated to horseradish peroxidase (GE Healthcare). The protein bands were probed with an ECL Enhanced chemiluminescence regent Kit (NCM Biotech), visualized using the MiniChemi™ 500 image system (Beijing Sage Creation, China) and analyzed by Image software (Lane 1D, Beijing Sage Creation, China).

### Immunoprecipitation and co-immunoprecipitation assays

In immunoprecipitation assays, RBMS1 stable knockdown or pLKO.1 control HEK293T cells were co-transfected with pCDH-PD-L1-3×FLAG and pCDH-HA-ubiquitin for 24 h using Sage LipoPlus reagent (Sage) according to the manufacturer’s instructions. Cells were collected and lysised using IP lysise buffer (50 mM Tris-HCl (pH 7.4), 150 mM NaCl, 1 mM EDTA, 1% TritonX-100, PMSF and Cocktail). The cell lysates were subjected to immunoprecipitation using anti-Flag M2 Affinity Gel (Sigma–Aldrich) at 4 ˚C overnight, followed by five times washes with 1 ml of washing buffer (50 mM Tris-HCl pH 7.5, 150 mM NaCl, 0.5% TritonX-100, 10% Glycerol). The affinity elute was analyzed by immunoblotting with anti-FLAG and anti-HA antibodies.

In co-immunoprecipitation assays, HEK293T cells were transfected with pCDH-PD-L1-3×FLAG (or pCDH-Flag empty vector) for 24 h using Sage LipoPlus reagent (Sage) according to the manufacturer’s instructions. Cells were lysised by IP lysise buffer (50 mM Tris-HCl (pH 7.4), 150 mM NaCl, 1 mM EDTA, 1% TritonX-100, PMSF and Cocktail). Cell lysates were incubated with anti-Flag M2 Affinity Gel (Sigma Aldrich) at 4 ˚C overnight. Following washes with 1 ml of washing buffer (50 mM Tris-HCl pH 7.5, 150 mM NaCl, 0.5% TritonX-100, 10% Glycerol), eluted protein samples was subjected to western blotting.

### RNA isolation and qRT-PCR

Total RNA from cultured cells was extracted using Trizol (Invitrogen) according to the manufacturer’s instructions. cDNAs were generated using Hifair^®^ II 1st Strand cDNA Synthesis SuperMix (Yeasen,China) with random primer and qPCR reactions were carried out using the Maxima SYBR Green qPCR Master Mix (Thermo Scientific). GAPDH mRNA were examined as an internal control for normalization. Gene expression changes relative to GAPDH were calculated using the ΔΔCT method.

### RNA-immunoprecipitation assay

RNA immunoprecipitation was carried out as previously described (Sheng, Zhao et al., 2018). In brief, Flag-RBMS1 or control cells were collected and cross-linked by 1% formaldehyde. Crosslinking reactions are blocked by the addition of glycine solution (pH 7.0) to a final concentration of 0.25 M for 5 min followed by two washes with ice-cold PBS. The cells resuspended in 1 mL of IP lysis buffer (50 mM Tris-HCl pH 7.5, 0.4 M NaCl, 1 mM EDTA, 1 mM DTT, 0.5% TritonX-100, 10% Glycerol containing protease inhibitors and RNase inhibitor) and subjected to three rounds of sonication. Solubilized cell lysate was precleared by mixing with Protein G-Sepharose beads along with nonspecific tRNA to get rid of non-specific binding. Then the precleared lysate were used for immunoprecipitation with Anti-Flag M2 Affinity beads. After five or six times washes with 1 mL of RIPA buffer (50 mM Tris-HCl pH 7.5, 0.4 M NaCl, 1 mM EDTA, 1 mM DTT, 0.5% TritonX-100, 10% Glycerol containing protease inhibitors and RNase inhibitor), the beads were resuspended in 100 μL RIP buffer (50 mM Tris-HCl pH 7.5, 0.1 M NaCl, 5 mM EDTA, 10 mM DTT, 0.5% TritonX-100, 10% Glycerol, 1% SDS) and incubated at 70 °C for 45 min to reverse the crosslinks. The RNA was extracted using Trizol and reverse transcribed into cDNA for PCR detection.

### Luciferase assay

The RBMS1 stable knockdown or pLKO.1 control MDA-MB-231 cells were plated in 96 well plates and the following day co-transfected with pGL3-B4GALT1-3′UTR luciferase reporters and Renilla plasmid for 24 h. The luciferase activities were measured following dual luciferase reporter assay detection kit (Promega Corporation, USA).

### T cell activation

Mouse lymphocytes were obtained from lymph nodes of BALB/c mice. The lymphocytes were activated by plates coated with 5 mg/mL anti-CD3 (145-2C11, Biolegend) and 2 mg/mL anti-CD28 (37.51, e-Bioscience), and cultured in RPMI-1640 medium plus 0.2% 2-mercaptoethanol (Gibco, 21985-023) and 2 mM L-Glutamine (Gibco, 21985-023) for 48 h. Jurkat cells were activated by plates coated with 1 μg/mL of CD3 antibody (OKT3, eBioscience) and 1 μg/mL of CD28 antibody (CD28.2, Invitrogen), and then cultured in RPMI1640 supplemented with 10% FBS for 48 h.

### Retrovirus production

Retroviral supernatants used for the transduction of human B7H3 specific CAR-T cells were prepared as previously described (Vera, Savoldo et al., 2006). Specifically, retrovirus with RD114 envelope was used.

### Transduction and expansion of human CAR-T cells

Peripheral blood mononuclear cells (PBMCs) from healthy donor were purchased from STEMCELL Technologies and were activated by plate coated with anti-CD3 and anti-CD28 mAbs. On day 2, T cells were transduced with retroviral supernatants using retronectin-coated plates (Takara Bio Inc.). On day 4, transduced T cells were collected from retronectin plate and expanded in complete T cell medium (90% RPMI-1640 (Hyclone), 10% FBS (Hyclone), 2 mM GlutaMAX, 100 unit/mL of Penicillin and 100 μg/mL of streptomycin) with IL-7 (10 ng/mL; PeproTech) and IL-15 (5 ng/mL; PeproTech), changing medium every 2–3 days as previously described (Ma, Shou et al., 2020). Functional assays were performed on day 10–14 post transduction.

### In vitro coculture assays

1 × 10^5^ tumor cells were seeded in 12-well plate in the presence of 10 ng/mL IFN-γ for 24 h. T cells (activated mouse T cells, activated Jurkat cells, or CAR-T cells) were then added to tumor cells at an indicated E/T ratio. Tumor cells not treated with T cells were used as control for T cell cytotoxicity. After 3 days, all cells in the well were collected and washed with PBS and analyzed by flow cytometry. Absolute cell count was measured by flow cytometry based on collection volume (30 uL).

### Flow cytometry

We performed flow cytometry using Abs specific to human CD7, CD45, B7-H3 and PD-L1, and murine CD8, CD45, PD-L1, IFN-γ and Granzyme B (from eBioscience and Biolegend) conjugated with FITC, PE, PE-cy7 and APC fluorochromes. For intracellular cytokine staining, lymphocytes were stimulated for 4 h with 50 ng/mL of PMA (phorbol 12-myristate 13-acetate) and 1 mM ionomycin in the presence of brefeldin A. Stained cells were analyzed on a Cytoflex S (Beckman Coulter) using CytExpert software or NovoCyte (Agilent) using NovoExpress software, and the flow data were analyzed with FlowJo software (v9.32, Tree Star).

### In vivo mice study

All animal experiments were performed according to protocols approved by the Institutional Animal Care and Use Committee (IACUC) of Dalian Medical University. For establishing tumor engraftment model, six-week-old Balb/c mice or nude mice were randomly and blindly divided into groups (five to six mice per group). Control sh, RBMS1 sh, or RBMS1 sh + B4GALT1 4T1 cells (2 × 10^5^ in 100 μL PBS) were subcutaneously injected into each mouse as indicated. Tumors were measured using a digital caliper and the tumor volume was calculated by the formula: (width)^2^ × length/2.

For establishing anti-CTLA4 therapeutic mouse model, control sh or RBMS1 sh 4T1 cells (2 × 10^5^ cells in 100 μl PBS) were subcutaneously injected into Balb/c mice. Mice were treatment with anti-CTLA4 (clone 9H10, 100 ug/mouse, i.p.) or isotype control antibody at day 6, 9, 12 and 15 following tumor cell inoculation.

The mice were euthanized before the longest dimension of the tumors reached 2.0 cm. Excised tumors were digested using Tumor Dissociation Kit (130-096-730, Miltenyi Biotec) at 37 °C for 45 min. The digested tissue was centrifuged in 40% / 80% percoll (Sigma) at 2000 rpm for 20 min to separate lymphocytes. Lymphocytes were then isolated and subjected to flow cytometry.

### Statistical analysis

All measurements were taken from distinct samples, as noted in figure legends, and no data were excluded. All data are presented as the means ± SD from at least three independent experiments. Sample sizes are noted in the figure legend. Statistical significance for each experiment was established by two-tailed unpaired or paired t test, and one-way or two-way ANOVA, as appropriate. Statistical analyses were performed using Prism 9 (Graph Pad) (ns, not significant; *p < 0.05, **p < 0.01, ***p < 0.001, ****p < 0.0001).

### Bioinformatics analysis

The proteomic data are obtained from TCGA breast cancer samples (Mertins et al., 2016). Subsequently, the breast cancer samples are grouped by their PAM50 subtypes. The genes are sorted based on the difference between the average proteomic level in the basal subtype and the average proteomic level across the other samples. The heat map was generated with r-package *ComplexHeatmap* (version 2.2.0) in R (version 3.6.3).

The GSE88847 dataset from the GEO database were retrieved from GEO website that included 38 patients with therapy-naïve TNBC. The TNBC subgroups (referred to as TIME subtypes) according to differential localizations of CD8 ^+^ T cells were requested from the author (Gruosso et al., 2019). Gene expression from the listed RNA binding proteins was processed in R (version 3.6.3). All software dependencies are available in the Comprehensive Repository R Archive Network (CRAN; https://cran.r-project.org/) or on Bioconductor (https://www.bioconducter.irg/).

### Study approval

The Institutional Animal Care and Use Committee of the Dalian Medical University approved use of animal models in this study. All human tumor tissues were obtained with written informed consent from patients or their guardians prior to participation in the study. The Institutional Review Board of the Dalian Medical University approved use of the tumor specimens in this study.

## Supplementary information

Supplemental Figure Legends

Supplemental Figure S1

Supplemental Figure S2

Supplemental Figure S3

Supplemental Figure S4

Supplemental Figure S5

Supplemental Figure S6

Supplementary Table 1

Supplementary Table 2

Supplemental Figure S7 Original uncut gel figures

signed Author contribution form

Reproducibility checklist

signed change of authorship request form

## Data Availability

RNA-seq data in this study have been deposited in Gene Expression Omnibus of NCBI with the accession code GSE183228. The authors declare that all the data supporting the findings of this study are available within the article and its Supplementary Information files.
