# Supplementary material for: Loss of RBMS1 promotes anti-tumor immunity through enabling PD-L1 checkpoint blockade in triple-negative breast cancer
Source: Cell Death Differ. 2022 May 10;29(11):2247–61. doi: 10.1038/s41418-022-01012-0 (PMC9613699; doi:10.1038/s41418-022-01012-0)
Supplement: Supplementary file 1 — Supplemental Figure Legends [file 41418_2022_1012_MOESM1_ESM.doc]

**SUPPLEMENTARY INFORMATION**

**Loss of** **RBMS1 promotes anti-tumor immunity through enabling PD-L1 checkpoint blockade in triple-negative breast cancer**

Jinrui Zhang^1#^, Ge Zhang^2#^, Wenjing Zhang^1#^, Lu Bai^1#^, Luning Wang^1^, Tiantian Li^1^, Li Yan^2, 3^, Yang Xu^3^, Dan Chen^4^, Wenting Gao^5^, Chuanzhou Gao^1^, Chaoqun Chen^1^, Menglin Ren^1^, Yuexia Jiao^1^, Hongqiang Qin^6^, Yu Sun^1^, Lili Zhi^1^, Yangfan Qi^1^, Jinyao Zhao^1^, Quentin Liu^1^, Han Liu^1^, Yang Wang^1*^

^1^Institute of Cancer Stem Cells and Second Affiliated Hospital, Dalian Medical University, Dalian 116044, China

^2^Department of Immunology, College of Basic Medical Sciences, Dalian Medical University, Dalian 116044, China

^3^School of Medicine, Southern University of Science and Technology, Shenzhen 518035, China

^4^Department of Pathology, First Affiliated Hospital, Dalian Medical University, Dalian 116044, China

^5^Institute of Genome Engineered Animal Models for Human Diseases, Dalian Medical University, Dalian 116044, China

^6^CAS Key Laboratory of Separation Science for Analytical Chemistry, Dalian Institute of Chemical Physics, Chinese Academy of Sciences, Dalian 116023, China.

^#^ These authors contributed equally to this work.

^*^ Correspondence:

Yang Wang; Institute of Cancer Stem Cells and Second Affiliated Hospital, Dalian Medical University, Dalian, China 116044; Tel: +86-411-86110531; Email: [yangwang@dmu.edu.cn](mailto:yangwang@dmu.edu.cn)

**Supplemental Figures Legends**

**Figure S1. Identification of RBMS1 as a key regulator of PD-L1. (A)** Proteomic analysis of 82 RNA binding proteins in TCGA samples of PAM50-defined intrinsic subtypes including 18 HER2, 29 Luminal A, 33 Luminal B, and 25 basal-like tumor samples. The genes are classified based on the difference between the average proteomic level in basal subtype and that of other breast cancer subtypes. **(B)** Heat map depicting expression of RNA binding proteins (mRNA) in the bulk tumor in FI (fully inflamed), SR (stroma restricted), MR (margin restricted), and ID (immune desert) TNBC (n = 38). **(C)** The mRNA levels of RBMS1 in FI, SR, MR, and ID were obtained from thirty-eight TNBC patient samples from GSE88847. (**D**) A western blot assay was utilized to determine the PD-L1 levels upon depletion of different RBPs in MDA-MB-231 cells. (**E**) The protein level of RBMS1 was examined in multiple breast cancer cell lines using a western blot assay. **(F)** The protein level of PD-L1 was examined with a western blot assay in mouse 4T1 breast cancer cells with stable depletion of RBMS1. **(G)** The protein level of PD-L1 was assayed in RBMS1 overexpressed MDA-MB-231, BT549, and HCC1937 breast cancer cells. **(H)** Cell surface analysis of PD-L1 protein was measured using flow cytometry in MDA-MB-231, BT-549, and HCC1937 breast cancer cells with stable overexpression of RBMS1. Data showed relative fold change in the MFI of PD-L1. Error bar represent mean ± SD. P values were determined by t-test. **(I)** 4T1 mouse breast cancer cells with stable depletion of RBMS1 were treated with or without IFN-γ. Cell surface analysis of PD-L1 protein using flow cytometry was shown. Data showed relative fold change in the MFI of PD-L1. Error bar represent mean ± SD. P values were determined by One-way ANOVA with Dunnett multiple comparisons. **(J)** The correlation of the protein levels of RBMS1 and PD-L1 from immunohistochemical staining in breast cancers (n = 40) and normal tissues (n = 10) were analyzed and plotted. For panels F, G, H, I, * P < 0.05, ** P < 0.01.

**Figure S2. Downregulation of RBMS1 promotes anti-tumor T cell immunity. (A)** A western blot assay was applied to examine the level of RBMS1 and PD-L1 in 4T1 mouse breast cancer cells with stable depletion of RBMS1. (**B**) The protein level of PD-L1 was measured in RBMS1 stably depleted MDA-MB-231, BT549, and HCC1937 cells. **(C)** T cell-mediated tumor cell killing assay in 4T1 cells with stable depletion of RBMS1. The quantitative ratio of dead cells is showed by the bar graph. Data represent mean ± SD, n = 3 independent repeats. P values were determined by One-way ANOVA with Dunnett multiple comparisons. ** P < 0.01, **** P < 0.0001.

**Figure S3. Depletion of RBMS1 promotes PD-L1 degradation via regulating glycosylation. (A)** The mRNA level of PD-L1 was examined with qRT-PCR assay in HCC1937, BT-549, and 4T1 cells with stable knockdown of RBMS1. NS denotes non-significant. **(B)** The mRNA level of PD-L1 was measured with qRT-PCR assay in MDA-MB-231 and BT-549 cells with overexpression of RBMS1. NS denotes non-significant. **(C)** RBMS1 stably depleted MDA-MB-231 cells were treated with 100 μg/mL cycloheximide (CHX) in the absence of IFN-γ treatment at the indicated time points. PD-L1 and RBMS1 levels were measured by immunoblotting. The intensity of PD-L1 was quantified and plotted. Three experiments were conducted with mean ± SEM presented. * P < 0.05**,** P values were determined by two-way repeated measures ANOVA. **(D)** Flag-PD-L1 was transiently transfected into RBMS1 stably depleted 293T cells in the absence of PS341. Then Flag-PD-L1 was immunoprecipitated by anti-FLAG M2-beads followed by immunoblot using antibody against ubiquitin. **(E)** RBMS1-depleted 293T cells were transiently transfected with wild-type PD-L1 or 4NQ-mutant-PD-L1 vector. The level of wild-type PD-L1 or 4NQ-mutant-PD-L1 was examined with a western blot approach. Data represent mean ± SD, n = 3 independent repeats. P values were determined using by One-way ANOVA with Dunnett multiple comparisons. * P < 0.05, ** P < 0.01, *** P < 0.001, **** P < 0.0001 for all panels.

**Figure S4. RBMS1 controls PD-L1 glycosylation via B4GALT1. (A)** The level of RBMS1 was examined in RBMS1-depleted MDA-MB-231 cells in the presence or absence of IFN-γ treatment. Data represent mean ± SD, n = 3 independent repeats. P values were determined by One-way ANOVA with Dunnett multiple comparisons. **(B)** The level of B4GALT1 and RBMS1 was examined in RBMS1-depleted BT549 cells using a qRT-PCR approach. Data represent mean ± SD, n = 3 independent repeats. P values were determined by unpaired t-test. **(C)** The levels of B4GALT1, B4GALT3, B4GALT4, B4GALT7, B3GNT3, and RBMS1 were examined in RBMS1-depleted MDA-MB-231 cells using a qRT-PCR approach. Data represent mean ± SD, n = 3 independent repeats. P values were determined by unpaired t-test. **(D)** The protein levels of B4GALT1 and RBMS1 were measured in RBMS1 stably overexpressed HCC1937 and BT549 cells using a western blot assay. **(E)** The protein levels of PD-L1 and B4GALT1 were examined in B4GALT1-depleted BT549 cells with or without the treatment of IFN-γ. **(F)** The protein levels of PD-L1 and B4GALT1 were examined in B4GALT1-overexpressed MDA-MB-231 and HCC1937 cells. **(G)** The protein levels of PD-L1, B4GALT1, and RBMS1 were measured in RBMS1-overexpressed BT549 cells with or without depletion of B4GALT1 in the presence of IFN-γ. (**H**) PLA was performed in MDA-MB-231 cells to examine the interaction between RBMS1 and B4GALT1. PLA signals were shown in red and the nuclei in blue. For (D-G), data represent mean ± SD, n = 3 independent repeats. P values were determined using by One-way ANOVA with Dunnett multiple comparisons. For panels A, B, *** P < 0.001, **** P < 0.0001.

**Figure S5. Depleted RBMS1 reduced the mRNA stability of B4GALT1.** (**A**) RBMS1-depleted BT549 cells were treated with actinomycin D as indicated times. The mRNA expression levels of B4GALT1 were examined using qRT-PCR. Error bars are mean ± SD from three biologically independent samples. P values were determined using Two-way repeated measures ANOVA. (**B**) Binding of *B4GALT1* 3′-UTR with RBMS1 is examined by RNA-immunoprecipitation in BT549 and HCC1937 cells expressing FLAG-RBMS1. **(C)** The protein level of RBMS1 was examined in RBMS1-depleted cells with or without re-expression of RBMS1. (**D**) The luciferase reporter B4GALT1-fluc-FL was transiently transfected into RBMS1 stably depleted 293T cells. The relative luciferase activities were determined by calculating the ratio of firefly luciferase activities over Renilla luciferase activities. The protein level of RBMS1 was examined. Error bars are mean ± SD from three biologically independent samples. P values were determined by One-way ANOVA with Dunnett multiple comparisons. (**E**) The luciferase reporters B4GALT1-fluc-T1 and B4GALT1-fluc-T2 were transiently transfected into RBMS1 stably depleted 293T cells. The relative luciferase activities were determined by calculating the ratio of firefly luciferase activities over Renilla luciferase activities. The protein level of RBMS1 was examined. Three independent experiments were conducted, with the mean ± SD of relative luciferase activities were shown. P values from a two-sided unpaired *t*-test. For panels A, D, E, * P < 0.05, ** P < 0.01, *** P < 0.001, **** P < 0.0001.

**Figure S6. Depleted RBMS1-stimulated anti-tumor T cell immunity is overturned by re-expression of B4GALT1.** **(A-B)** T cell-mediated tumor cell killing assay in MDA-MB-231 (A) and 4T1 (B) cells with stable depletion of RBMS1 with or without re-expression of B4GALT1. The quantitative ratio of dead cells is showed by the bar graph. Data represent mean ± SD, n = 3 independent repeats. P values were determined by One-way ANOVA with Tukey’s multiple comparisons. * P < 0.05, ** P < 0.01.
