# Supplementary figures and images for: Loss of RBMS1 promotes anti-tumor immunity through enabling PD-L1 checkpoint blockade in triple-negative breast cancer
Source: Cell Death Differ. 2022 May 10;29(11):2247–61. doi: 10.1038/s41418-022-01012-0 (PMC9613699; doi:10.1038/s41418-022-01012-0)

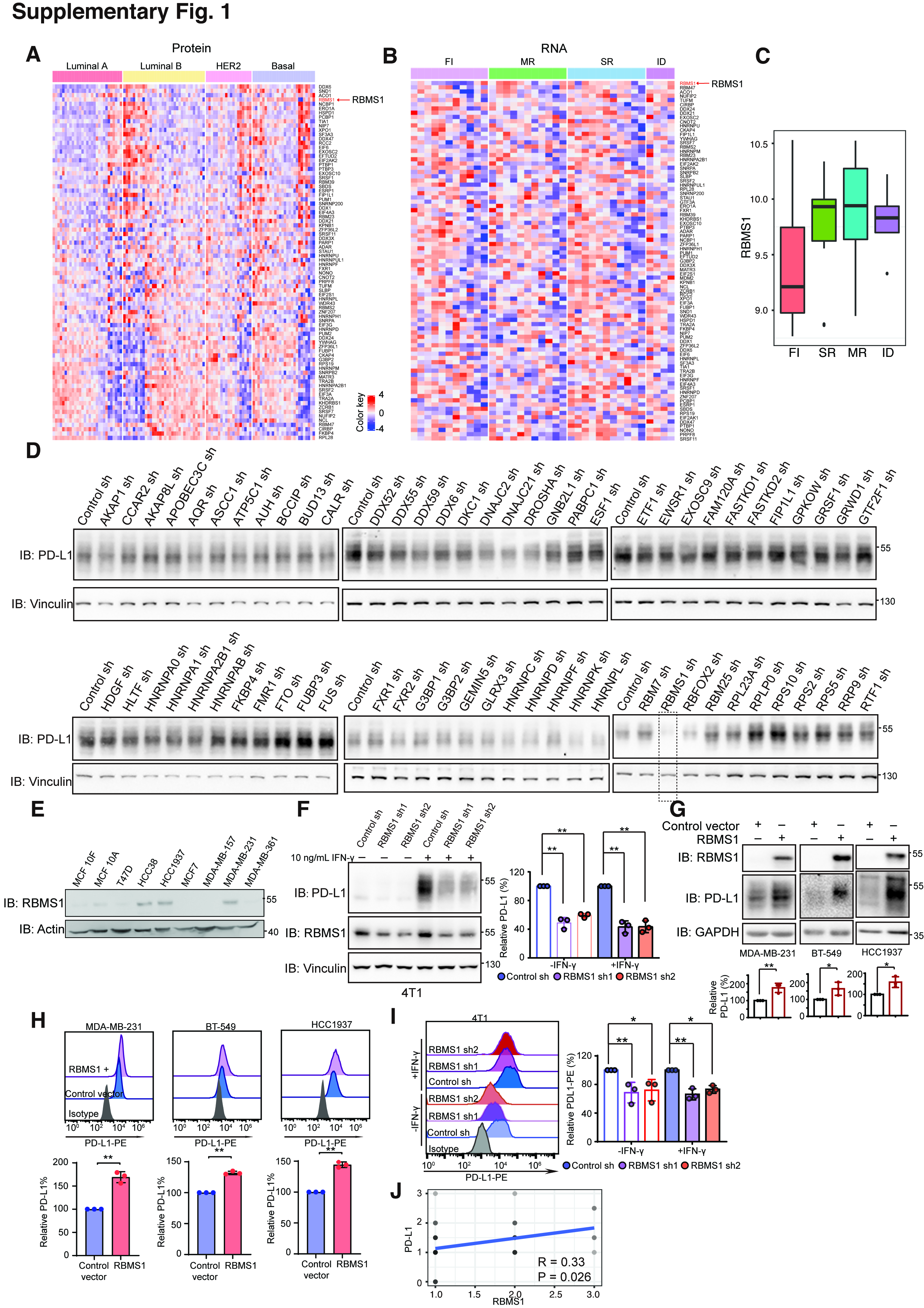

Supplement: Supplementary file 2 — Supplemental Figure S1 [file 41418_2022_1012_MOESM2_ESM.tif]

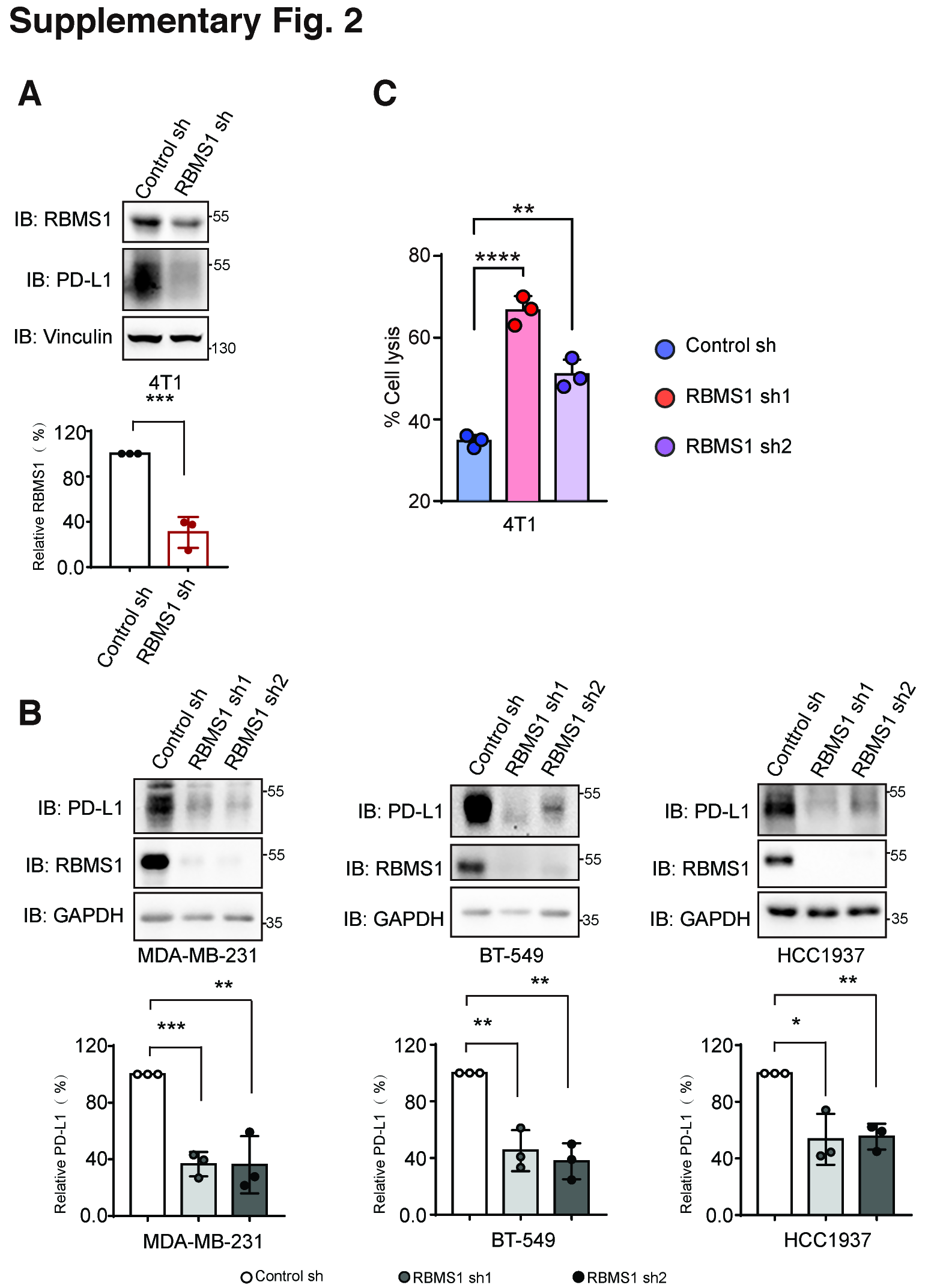

Supplement: Supplementary file 3 — Supplemental Figure S2 [file 41418_2022_1012_MOESM3_ESM.tif]

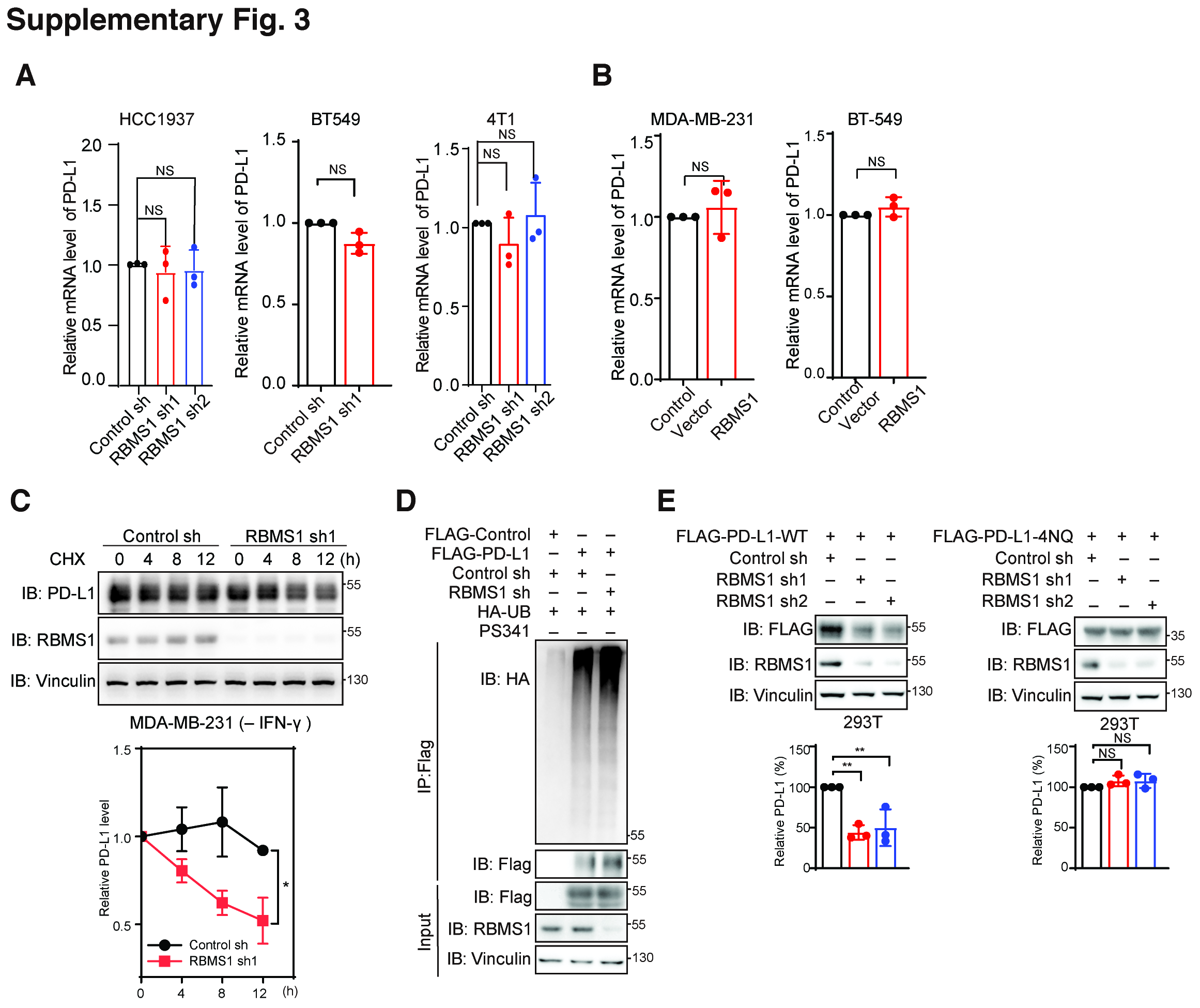

Supplement: Supplementary file 4 — Supplemental Figure S3 [file 41418_2022_1012_MOESM4_ESM.tif]

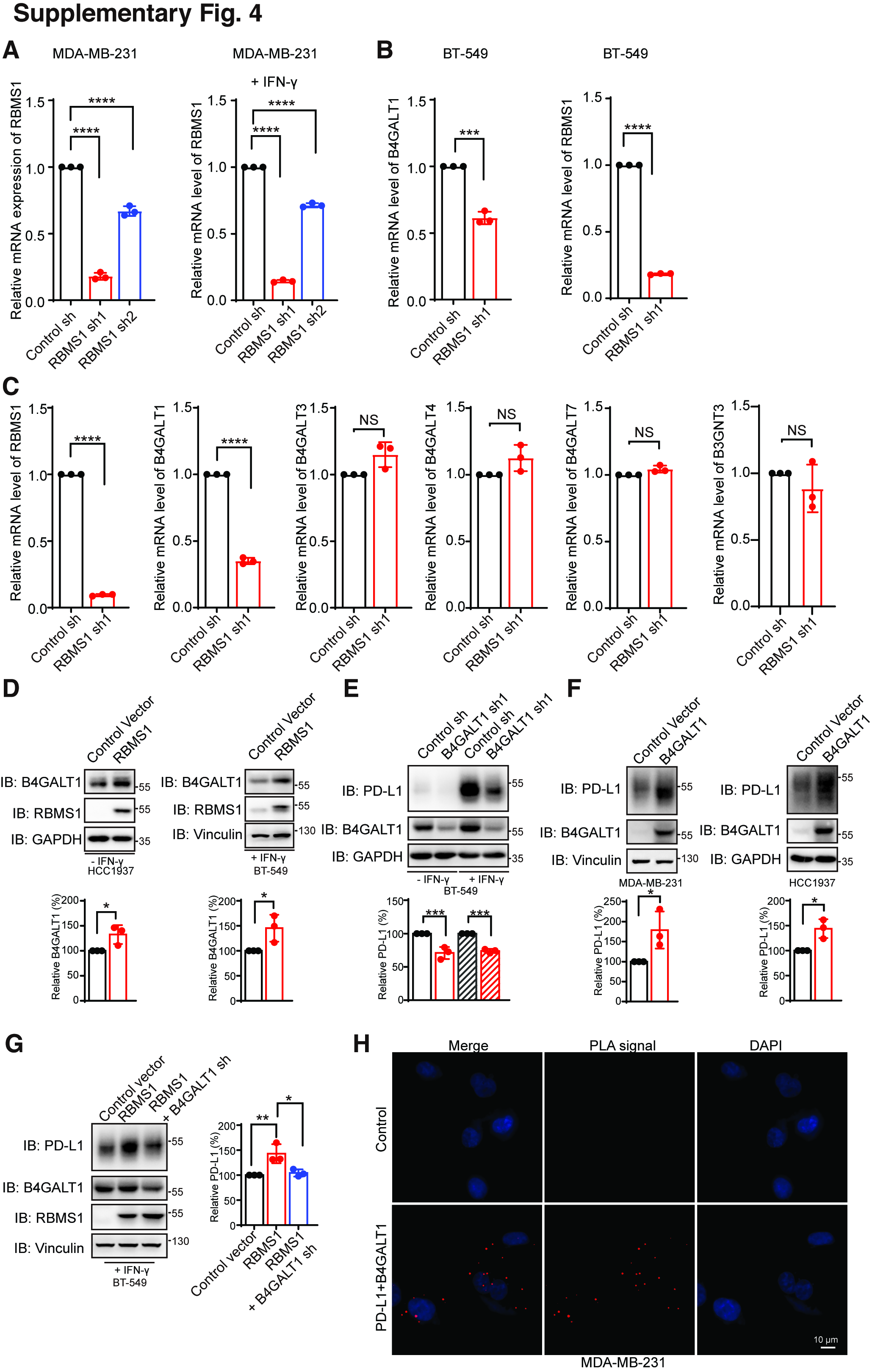

Supplement: Supplementary file 5 — Supplemental Figure S4 [file 41418_2022_1012_MOESM5_ESM.tif]

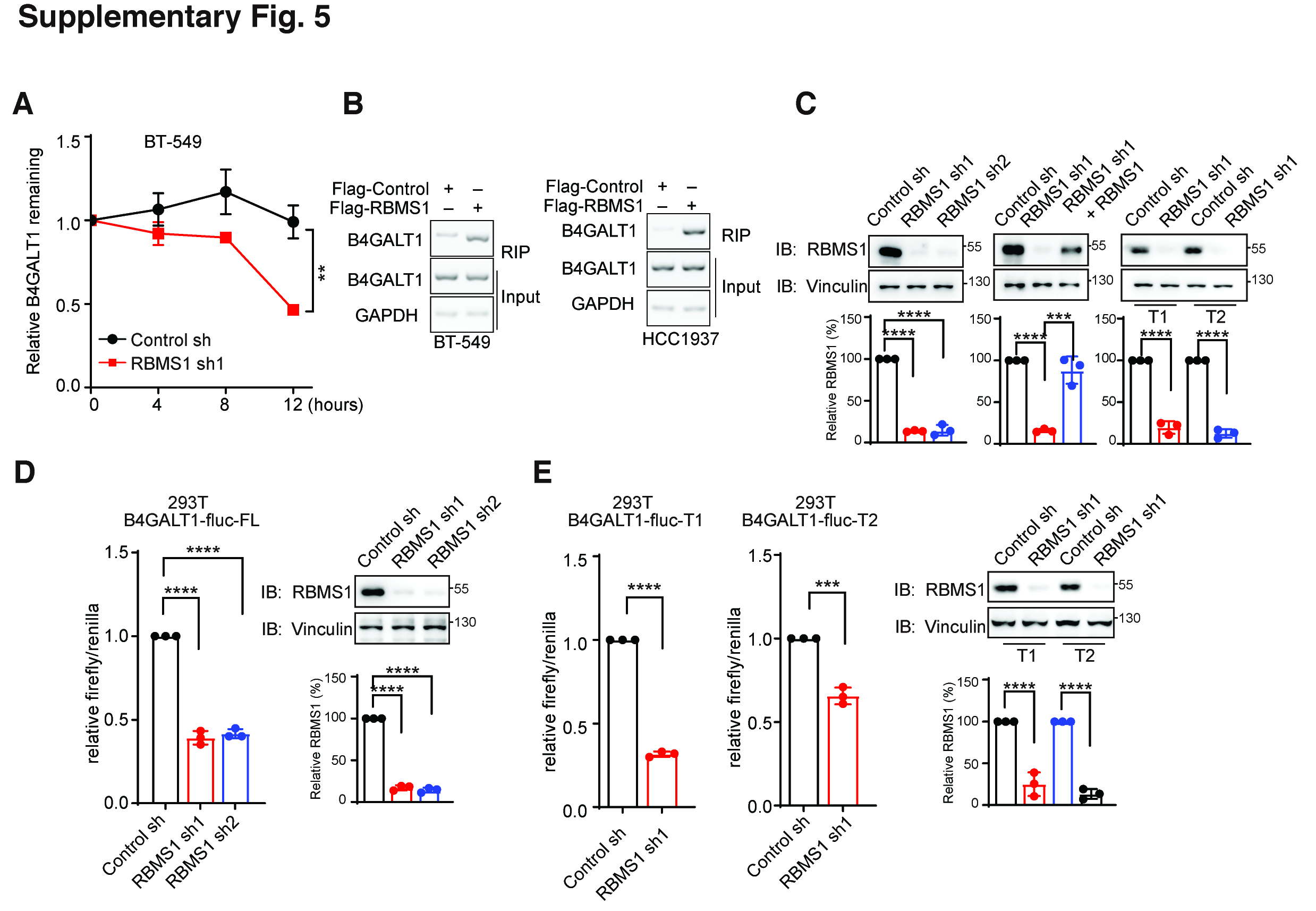

Supplement: Supplementary file 6 — Supplemental Figure S5 [file 41418_2022_1012_MOESM6_ESM.tif]

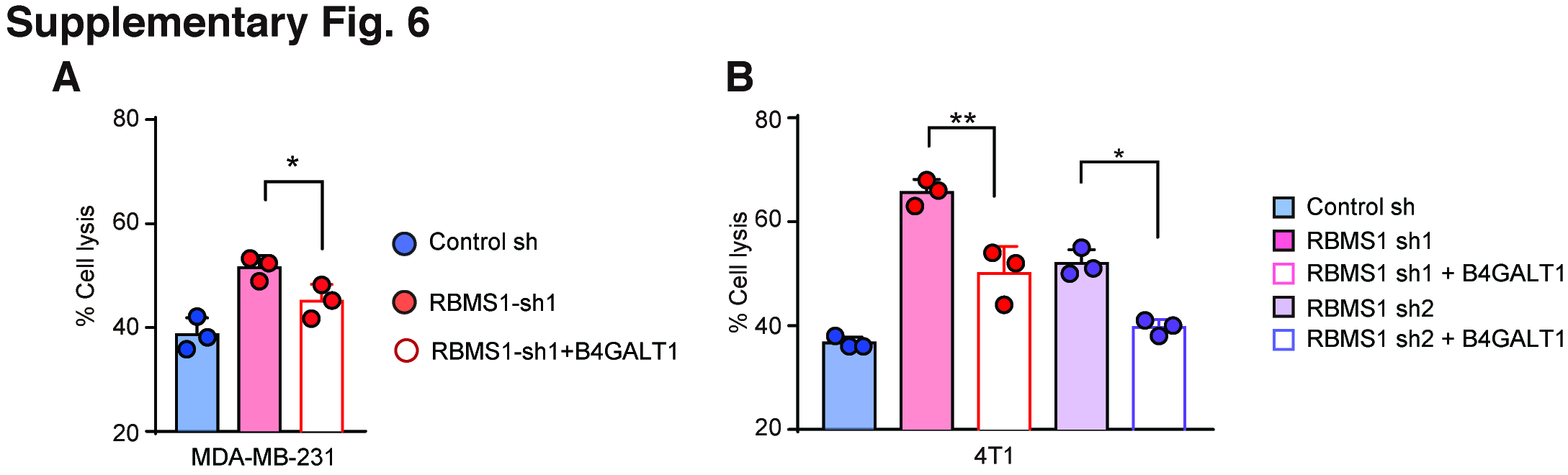

Supplement: Supplementary file 7 — Supplemental Figure S6 [file 41418_2022_1012_MOESM7_ESM.tif]
