# Supplementary material for: Loss of RBMS1 promotes anti-tumor immunity through enabling PD-L1 checkpoint blockade in triple-negative breast cancer
Source: Cell Death Differ. 2022 May 10;29(11):2247–61. doi: 10.1038/s41418-022-01012-0 (PMC9613699; doi:10.1038/s41418-022-01012-0)

Figure.1E

MDA-MB-231

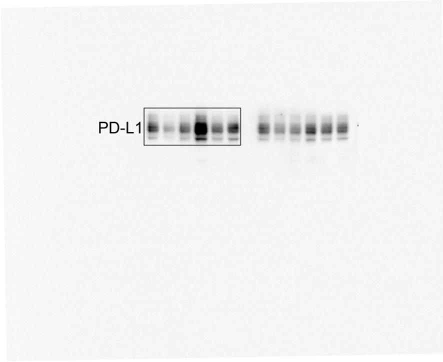

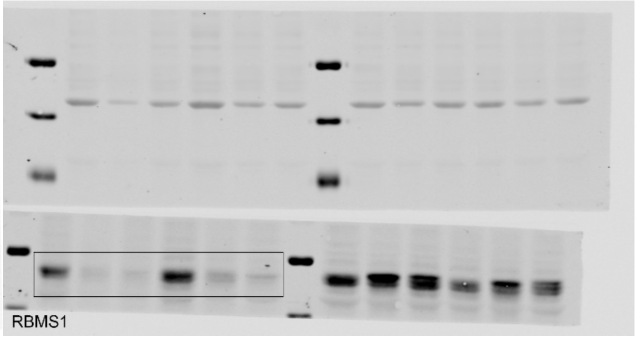

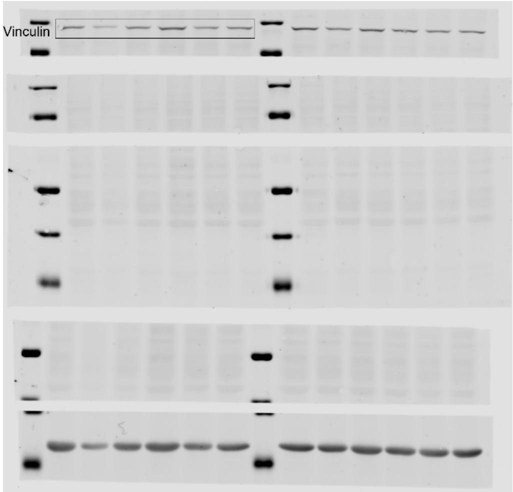

HCC1937

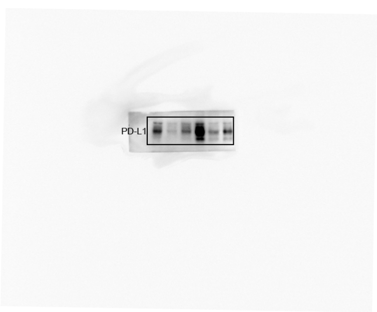

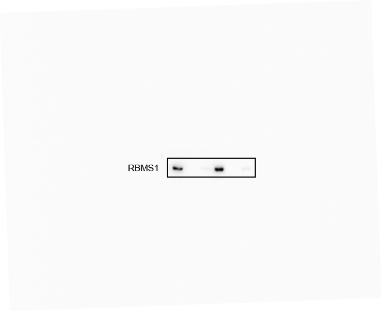

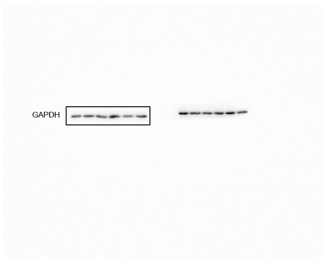

BT-549

-IFNγ

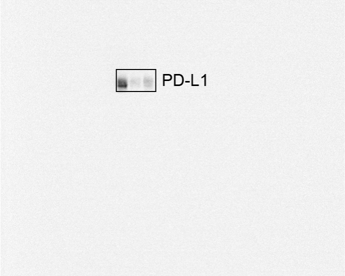

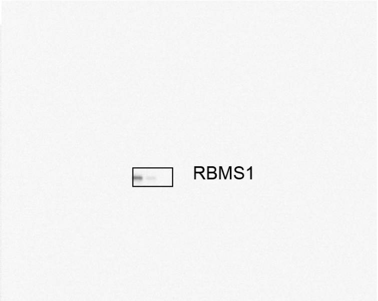

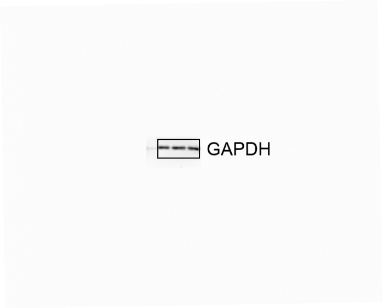

+IFNγ

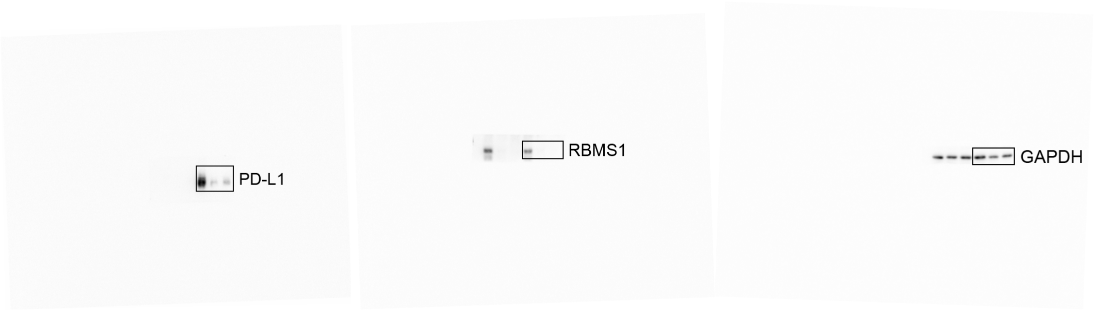

Figure.3B

MDA-MB-231

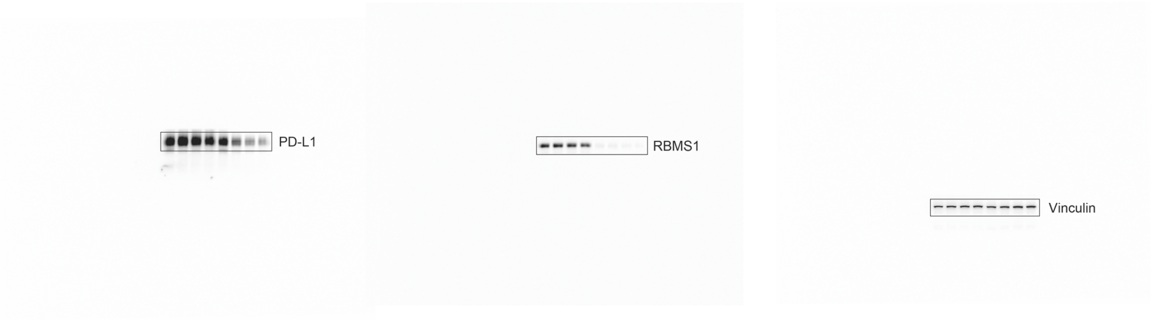

4T1

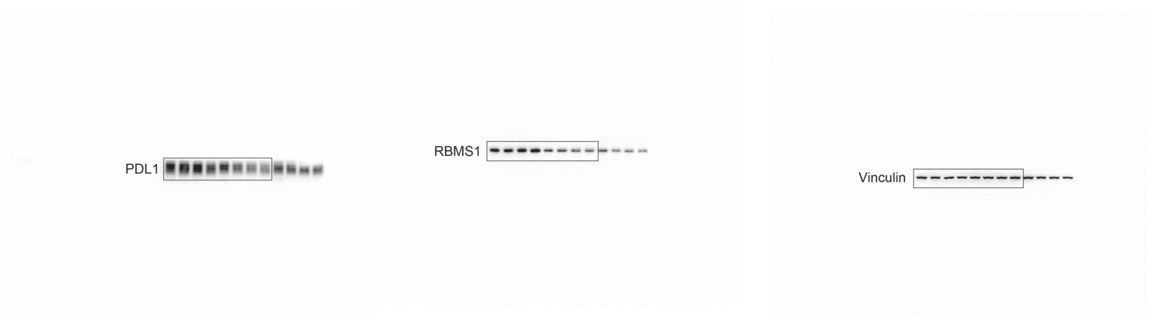

Figure.3C

MDA-MB-231

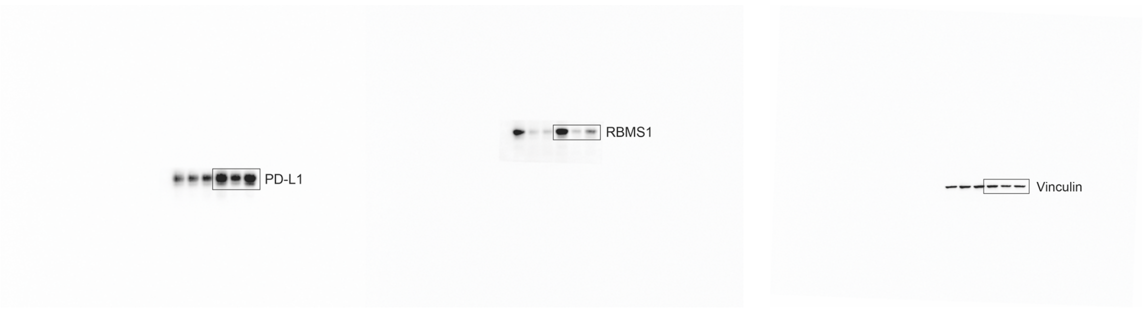

Figure.3D
HEK-293T

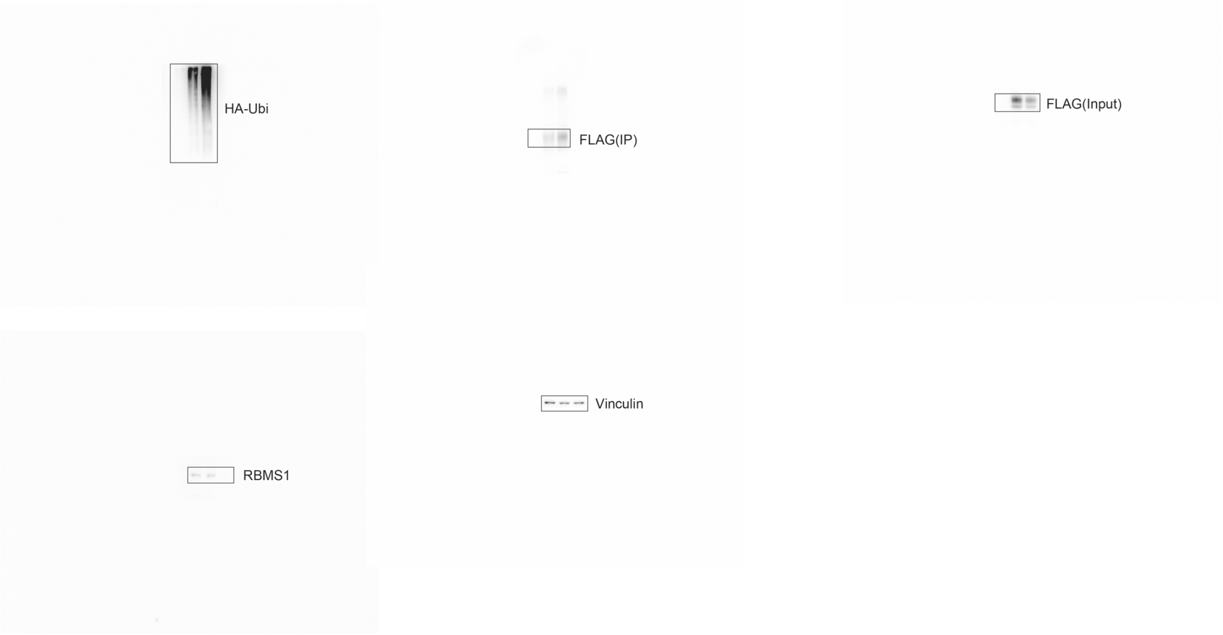

Figure.3E
MDA-MB-231

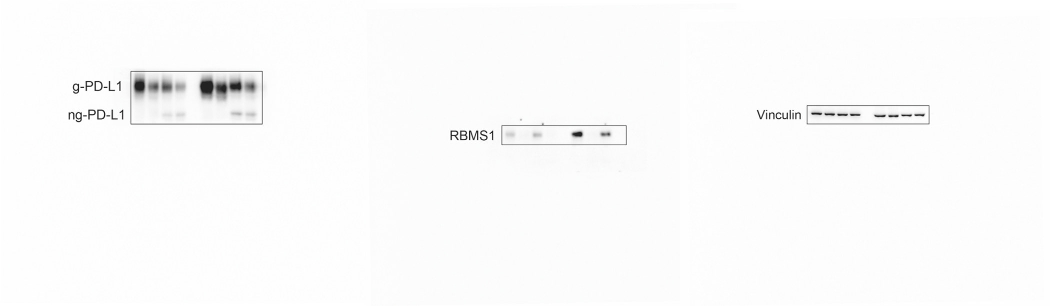

Figure.3F
MDA-MB-231

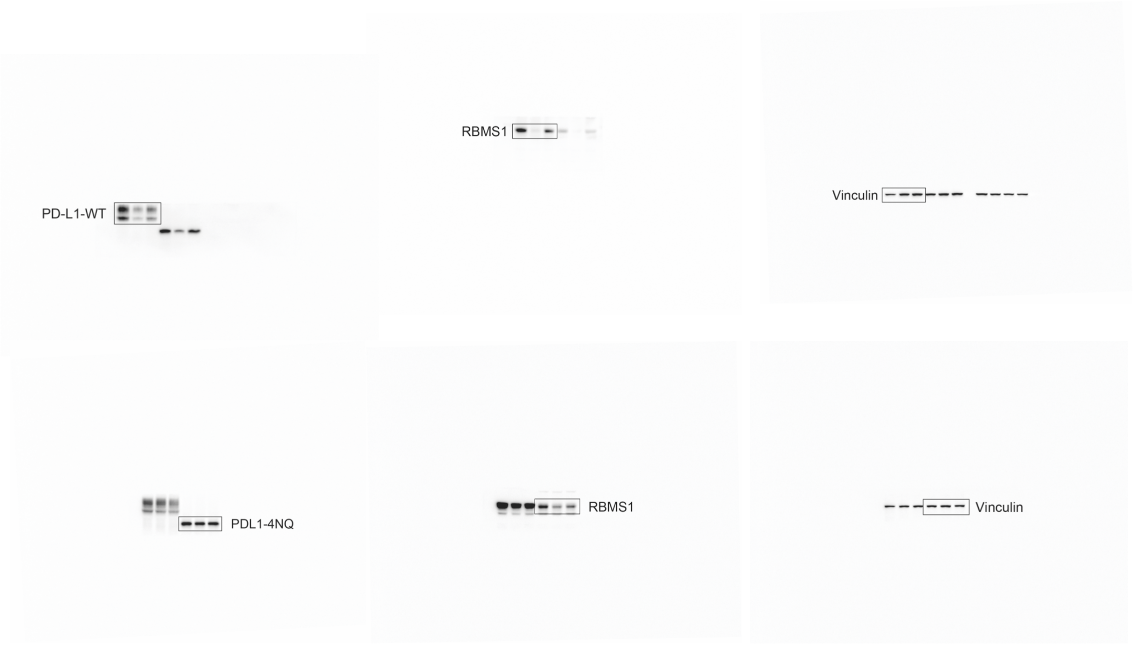

Figure.4B

MDA-MB-231

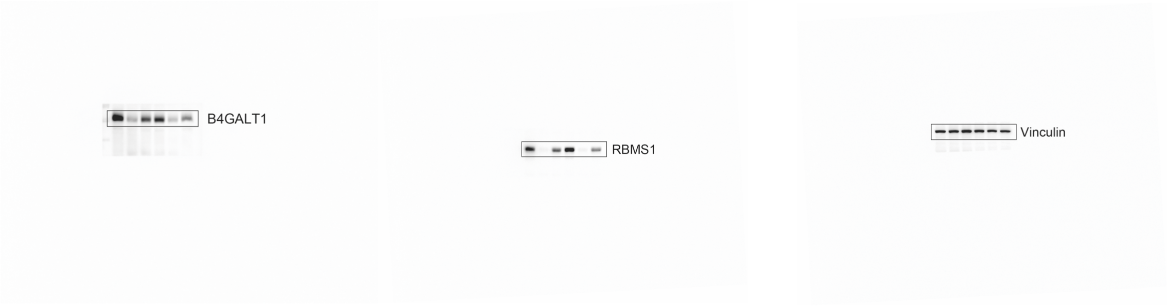

BT-549

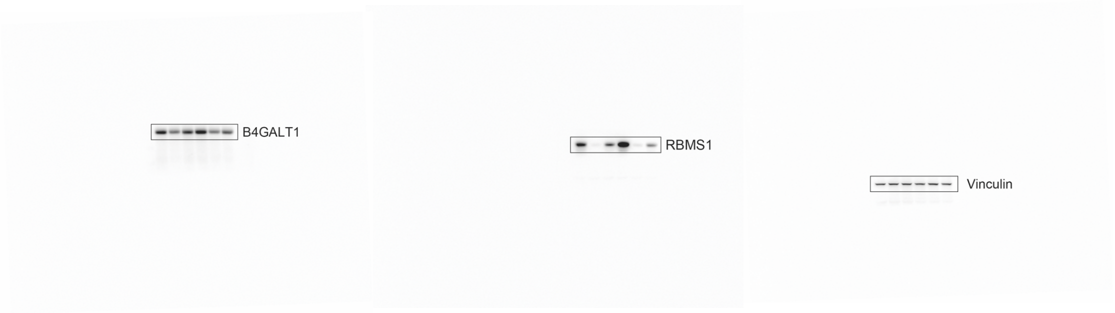

Figure.4D

MDA-MB-231

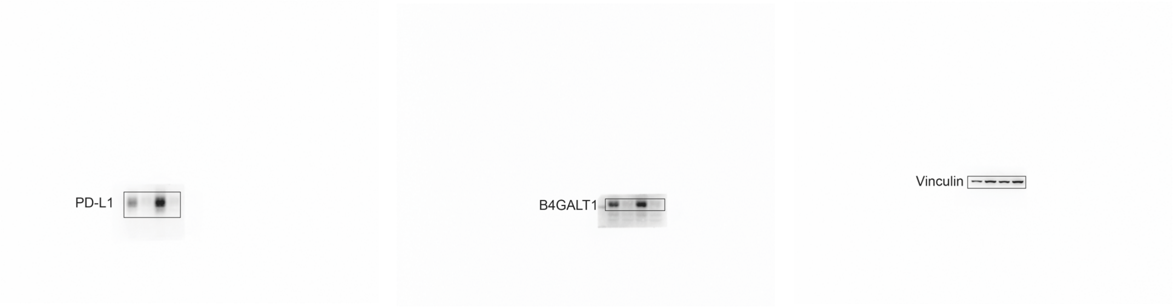

HCC1937

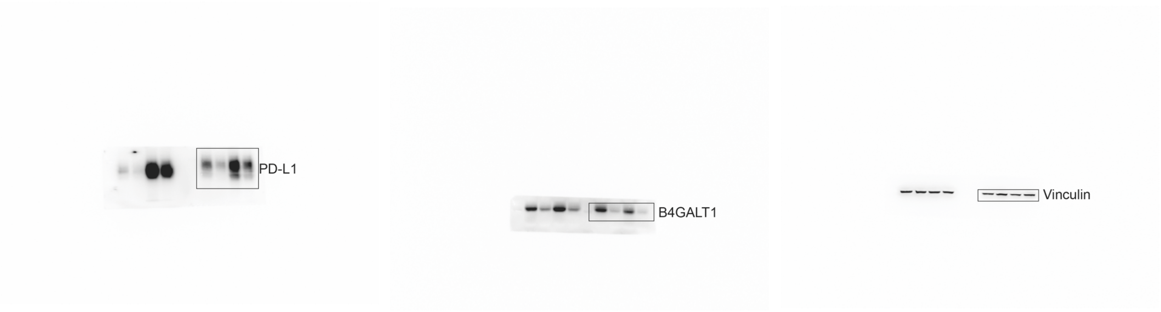

Figure.4E

MDA-MB-231

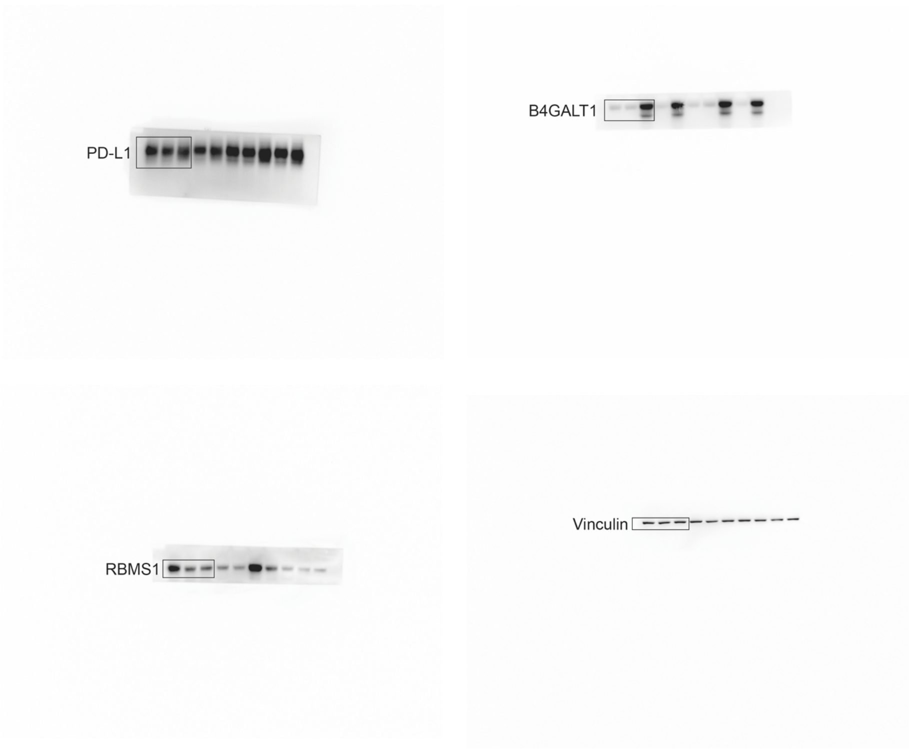

BT-549

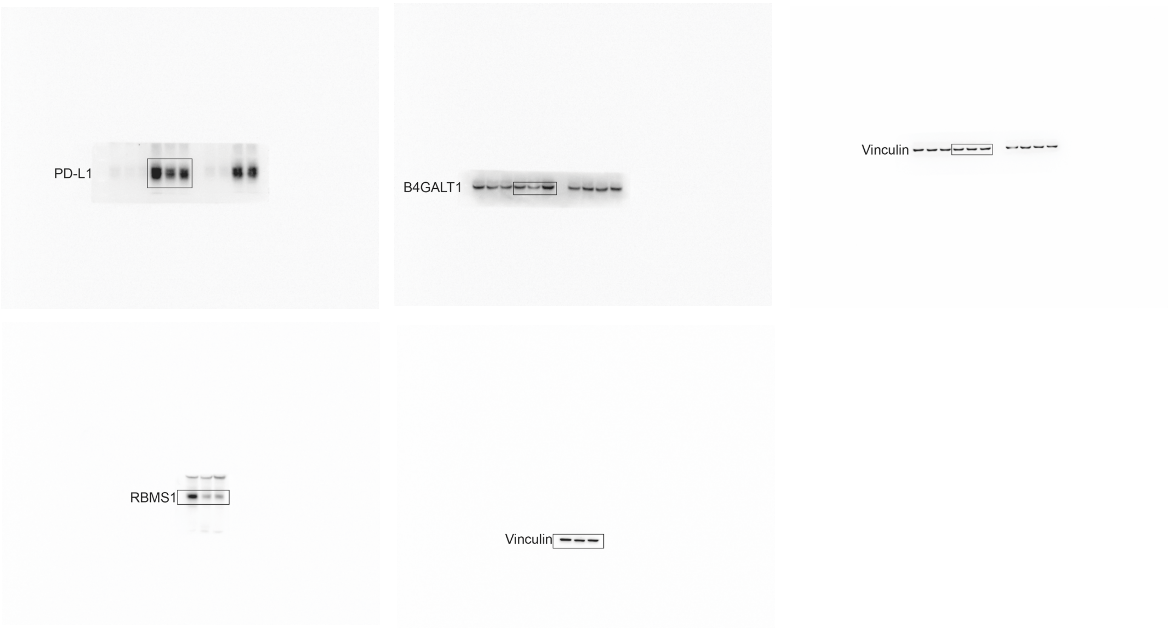

Figure.4F
MDA-MB-231

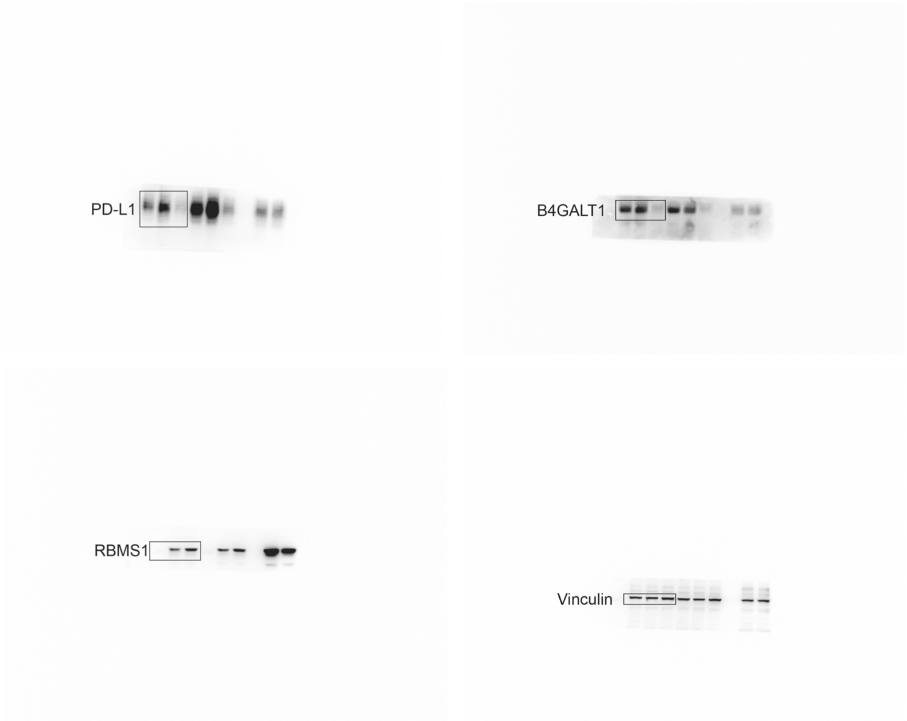

Figure.4G
HEK-293T

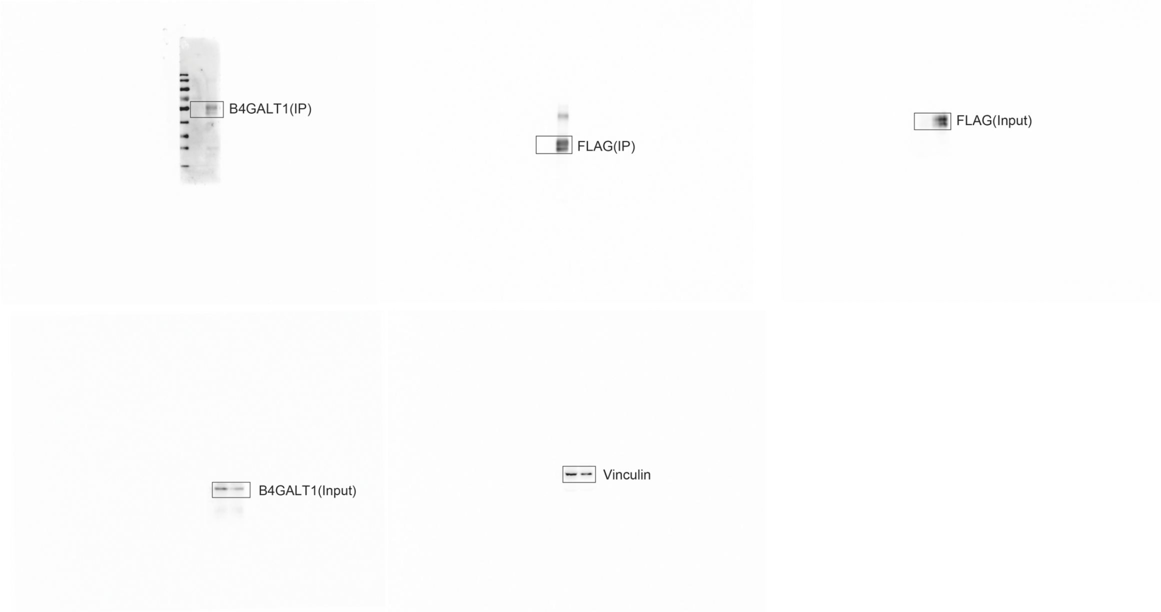

HEK-293T

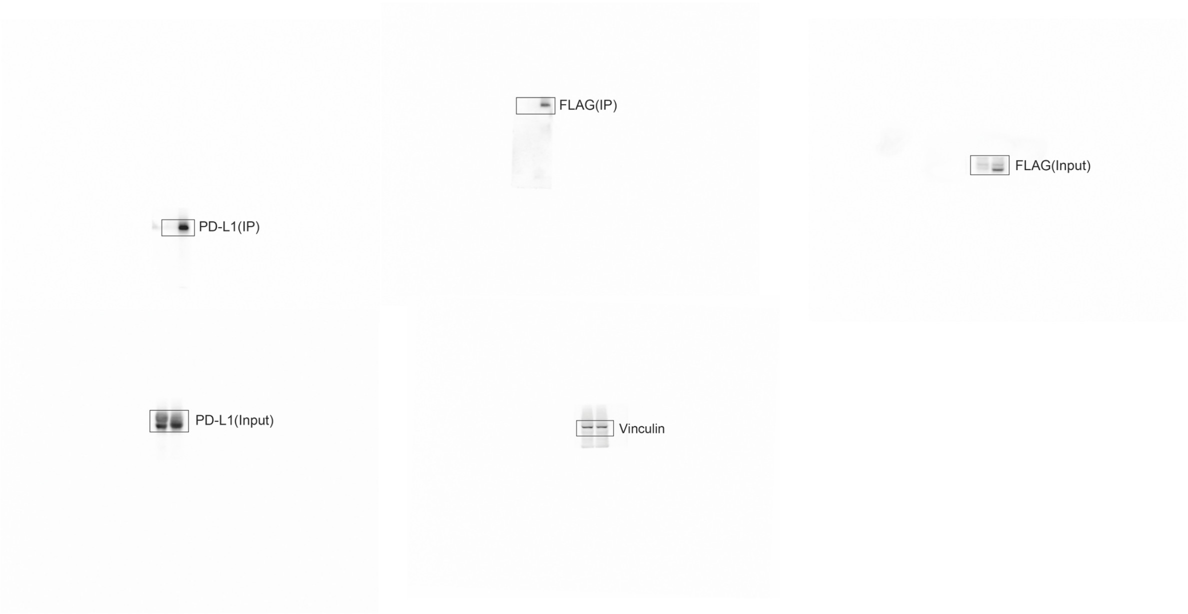

Supplementary Fig.1D

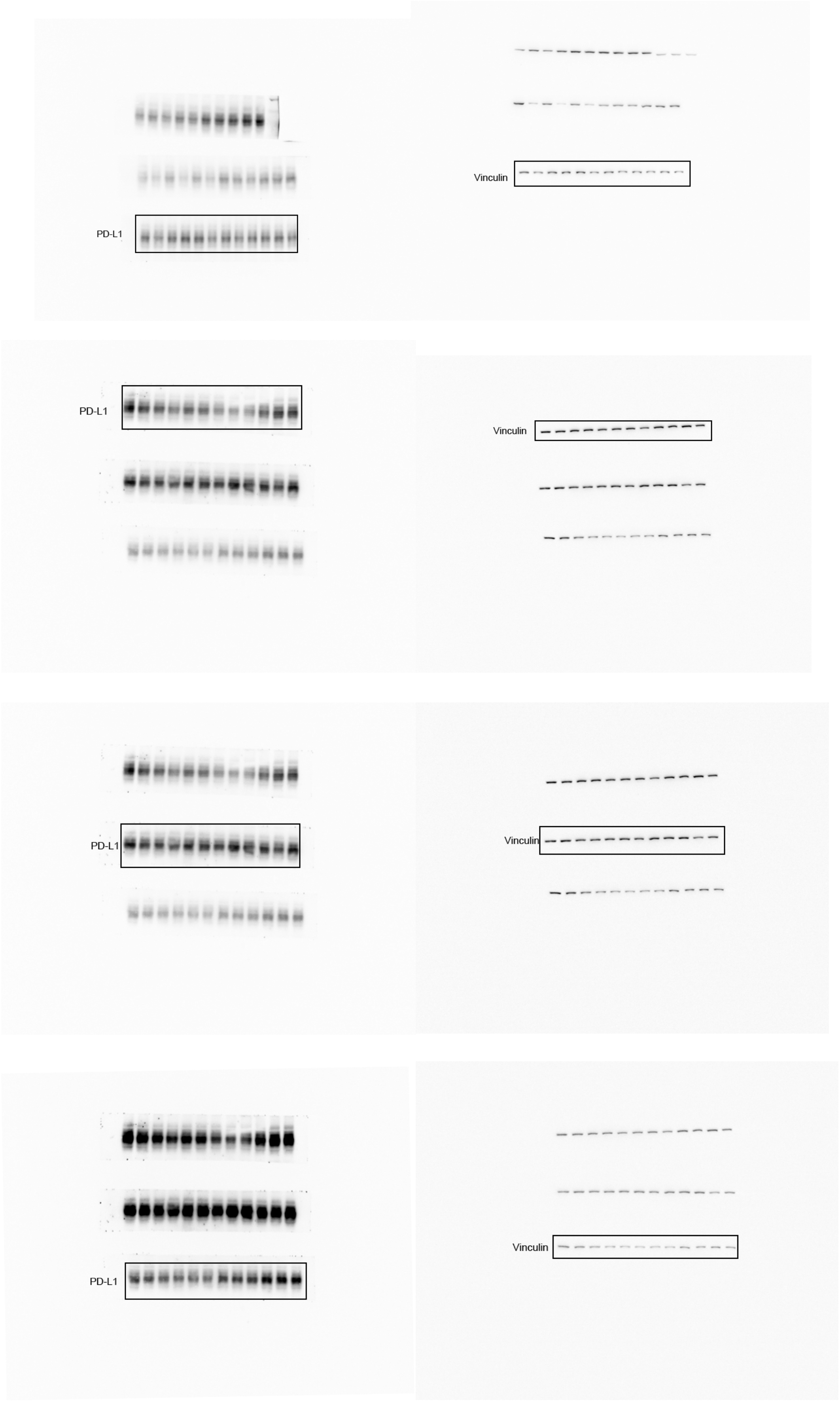

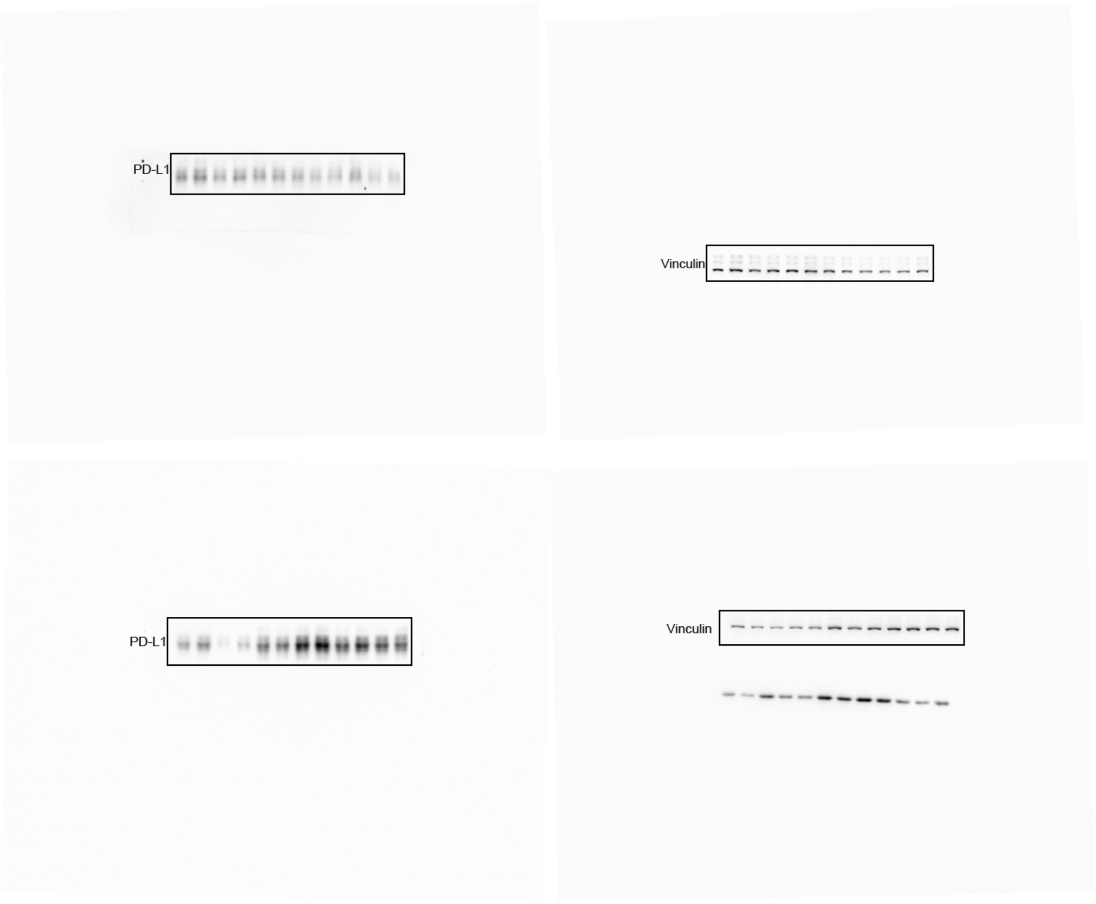

Supplementary Fig.1E

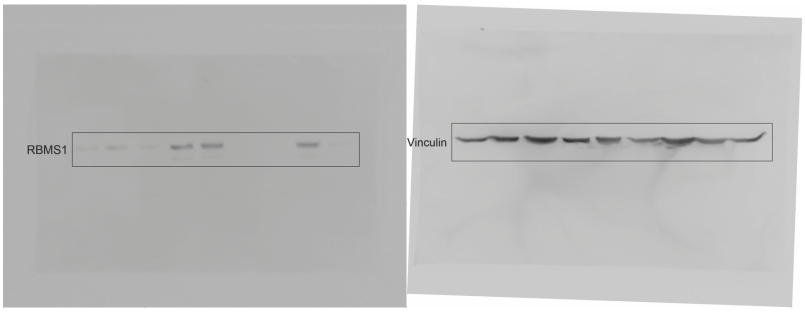

Supplementary Fig.1F

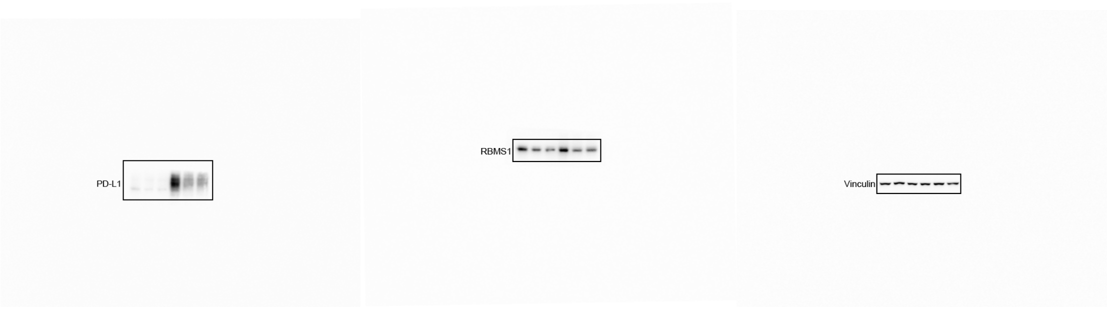

Supplementary Fig.1G

MDA-MB-231

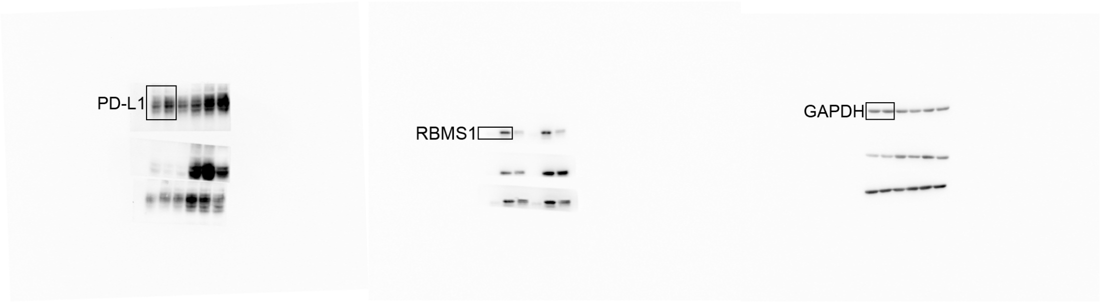

BT-549

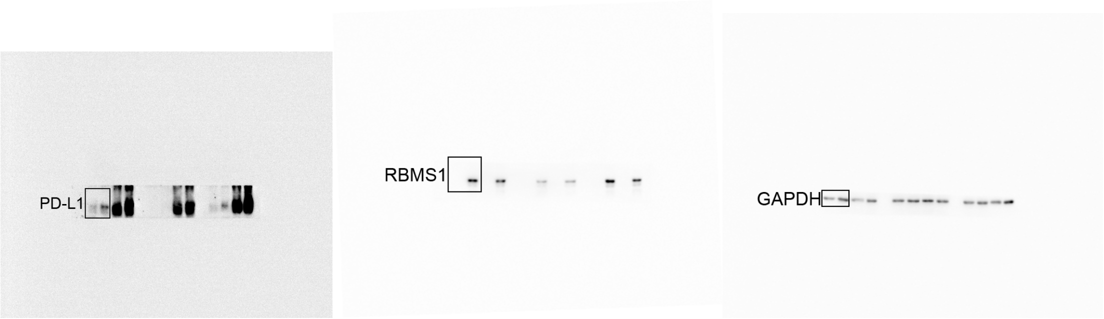

HCC1937

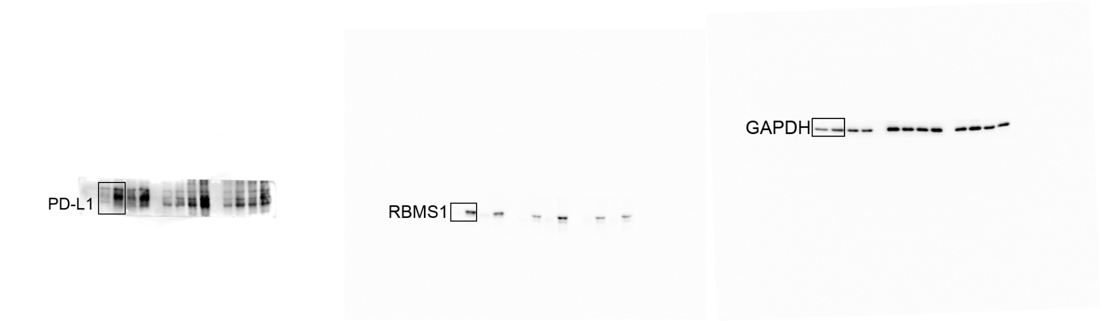

Supplementary Fig.2A

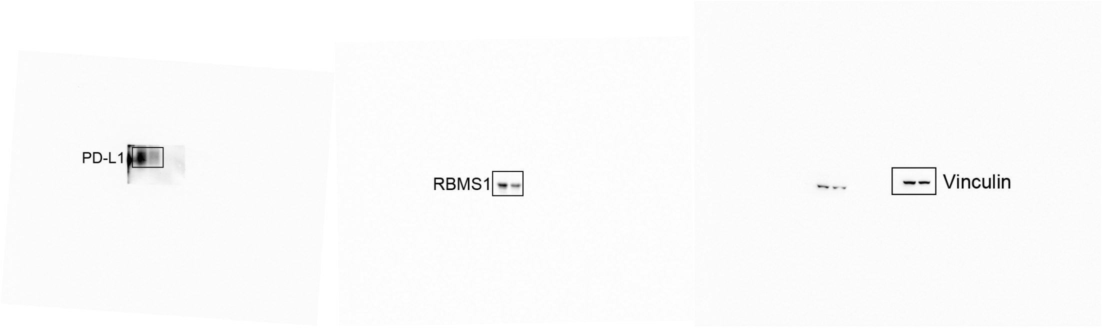

Supplementary Fig.2C

MDA-MB-231

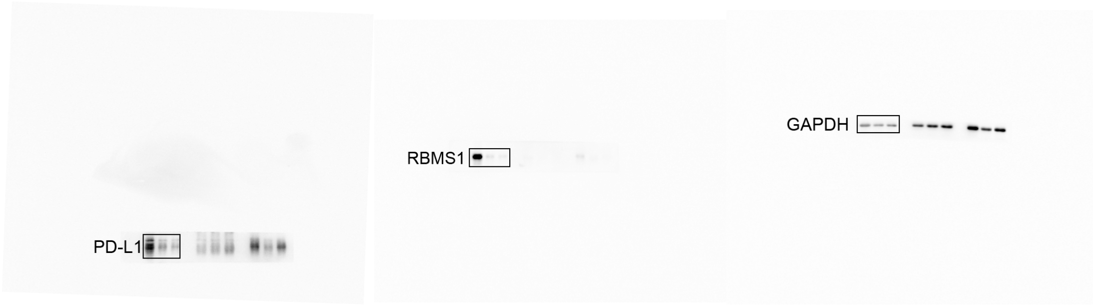

BT-549

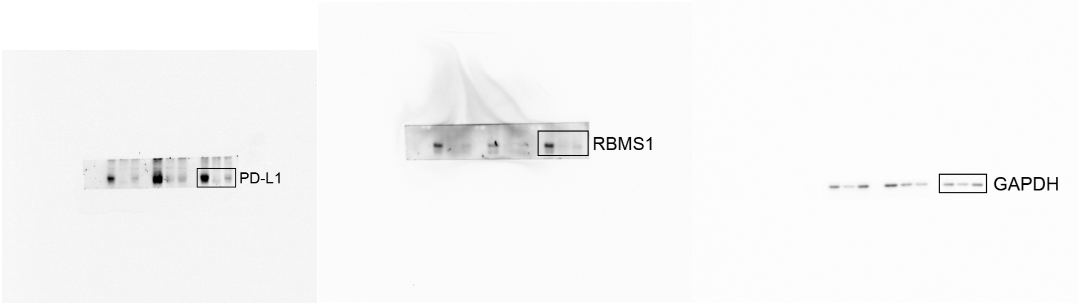

HCC1937

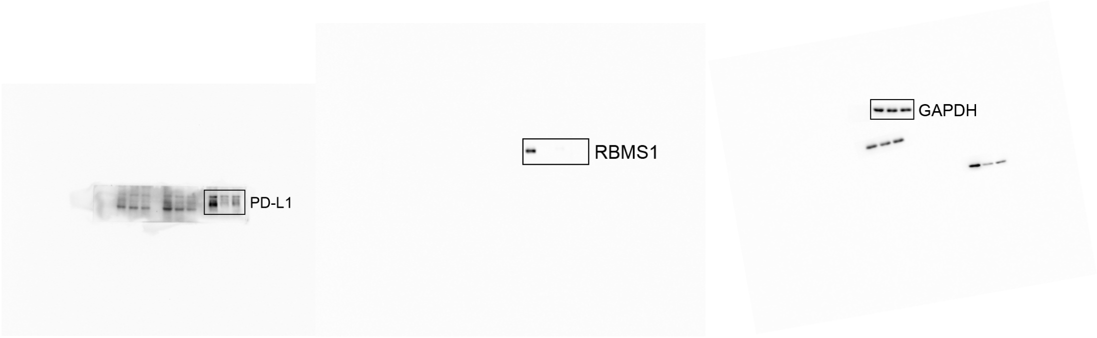

Supplementary Fig.3C
MDA-MB-231

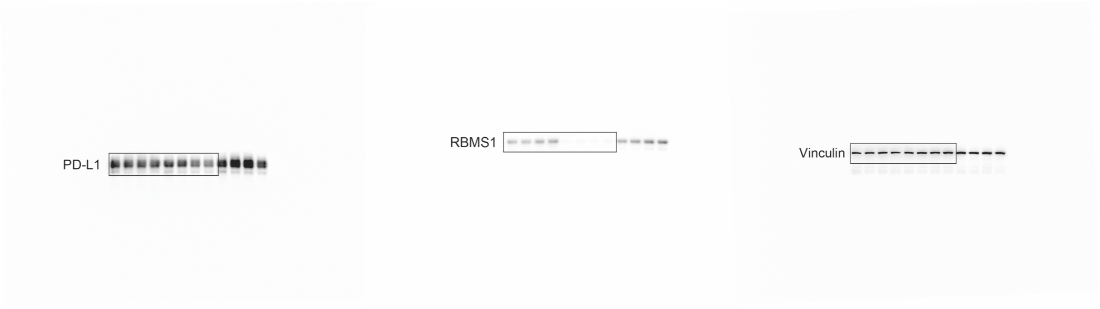

Supplementary Fig.3D
HEK-293T

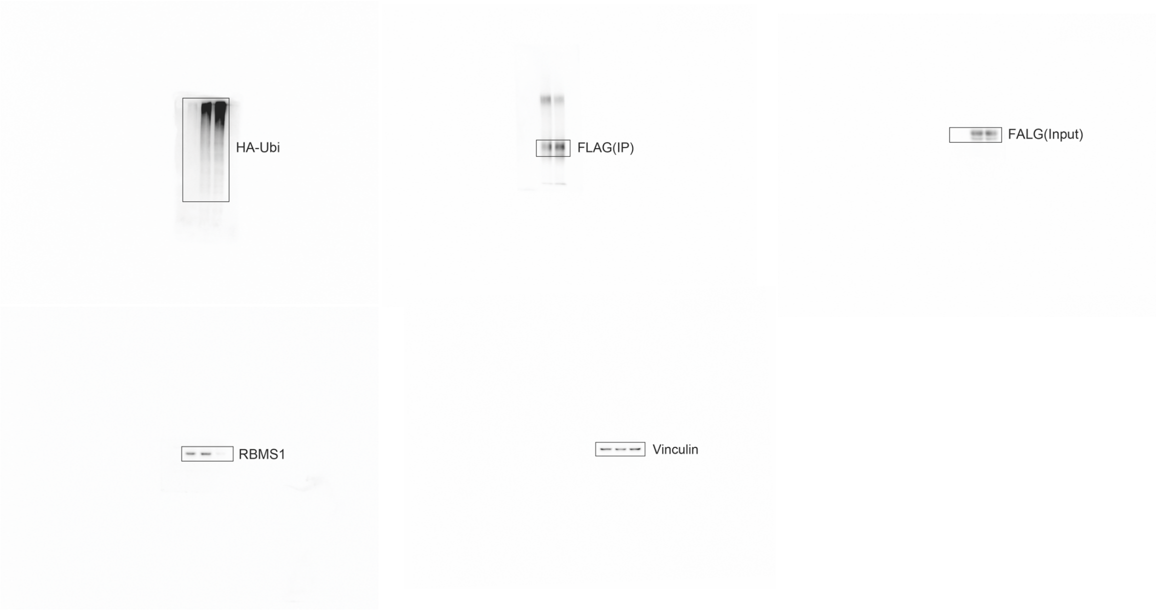

Supplementary Fig.3E
HEK-293T

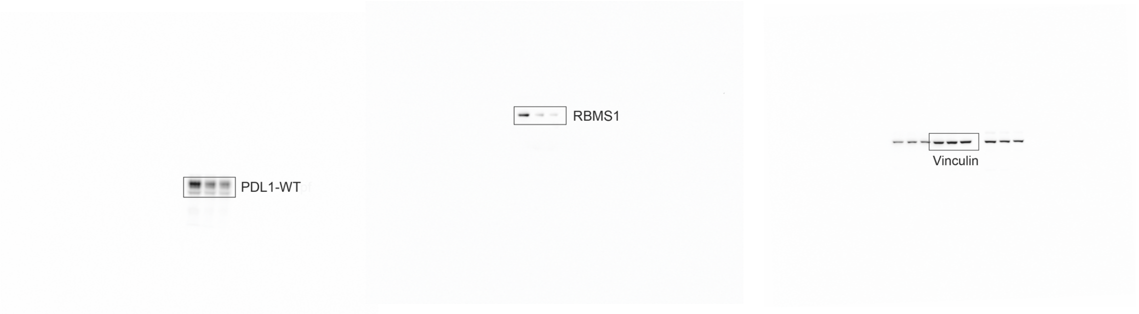

HEK-293T

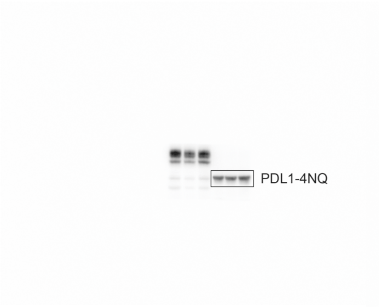

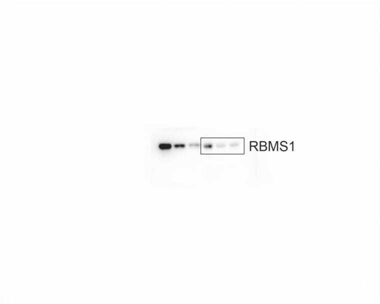

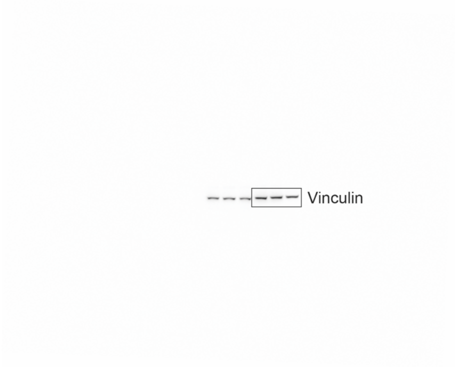

Supplementary Fig.4D

HCC1937

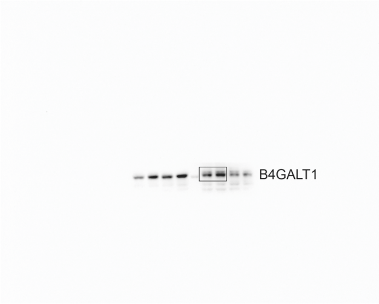

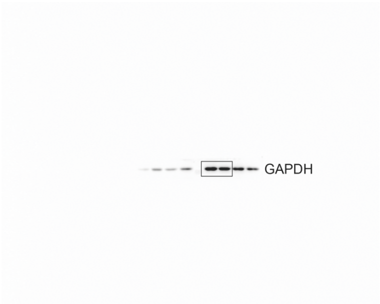

BT-549

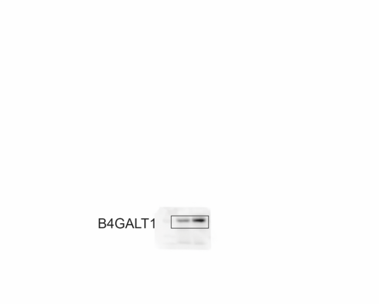

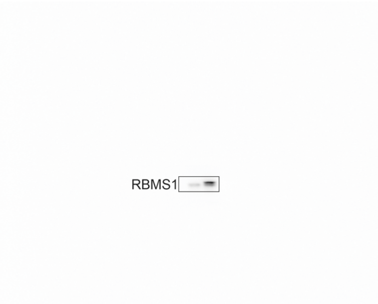

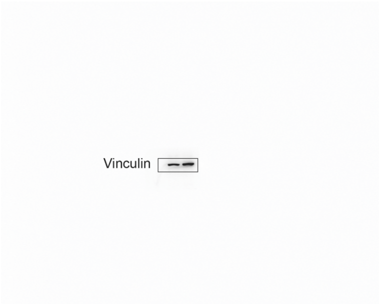

Supplementary Fig.4E

BT-549

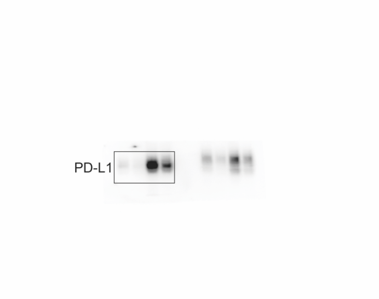

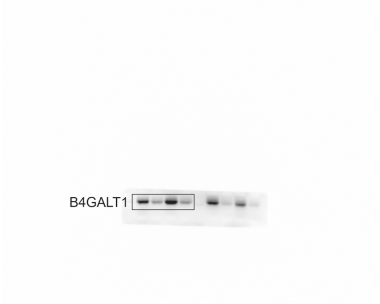

Supplementary Fig.4F

MDA-MB-231

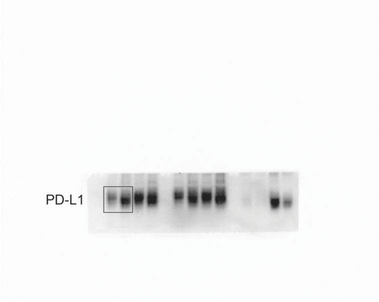

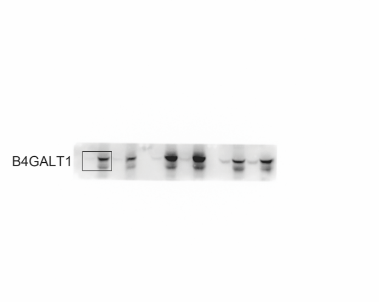

HCC1937

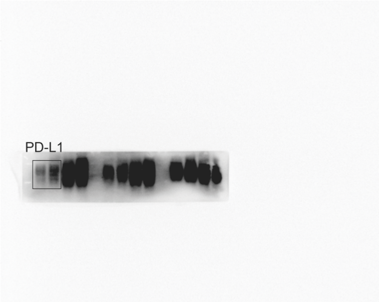

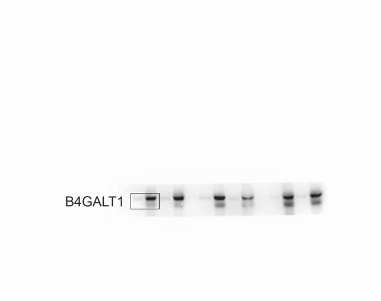

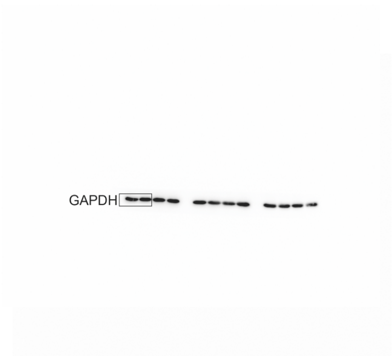

Supplementary Fig.4G

BT-549

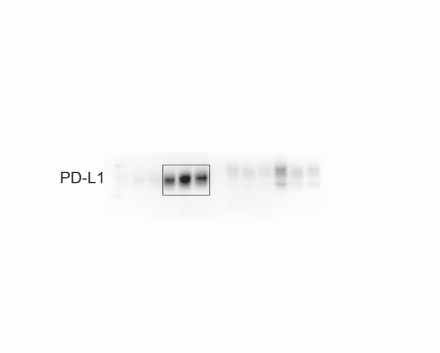

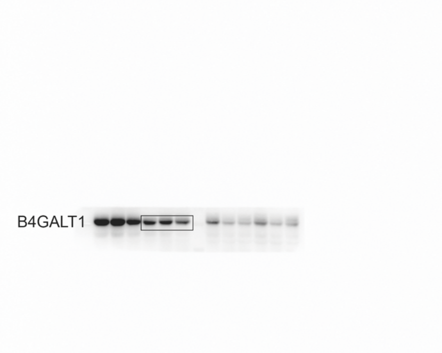

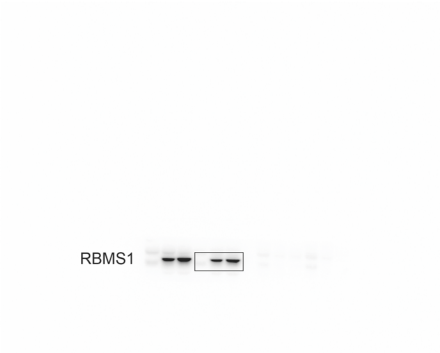

Supplementary Fig.5C
MDA-MB-231

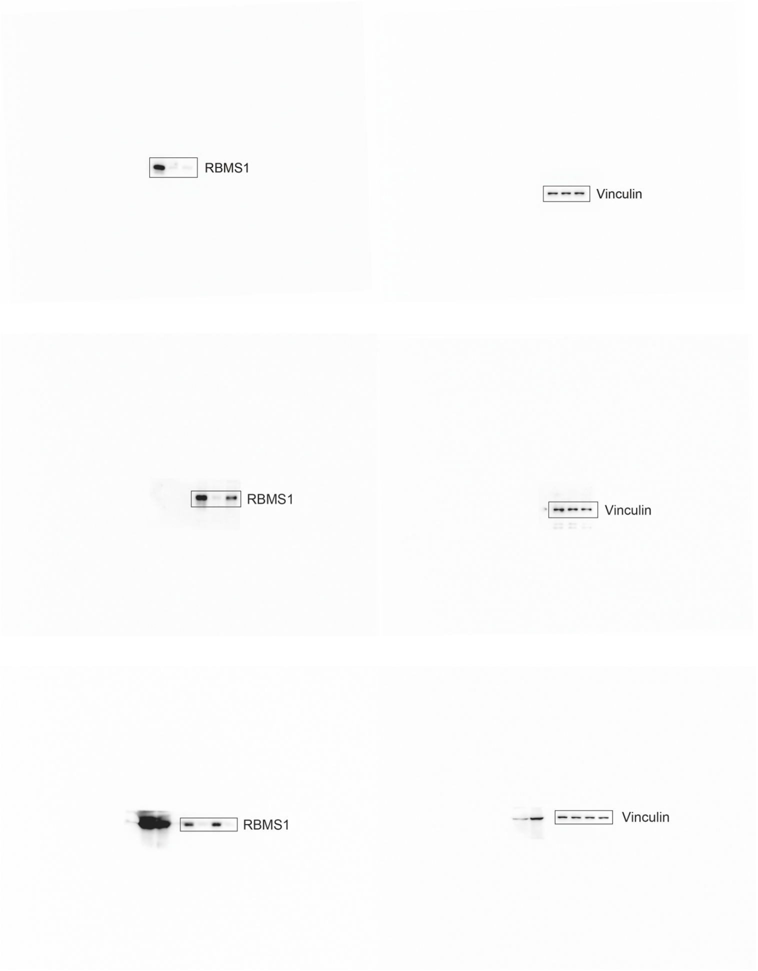

Supplementary Fig.5D
HEK-293T

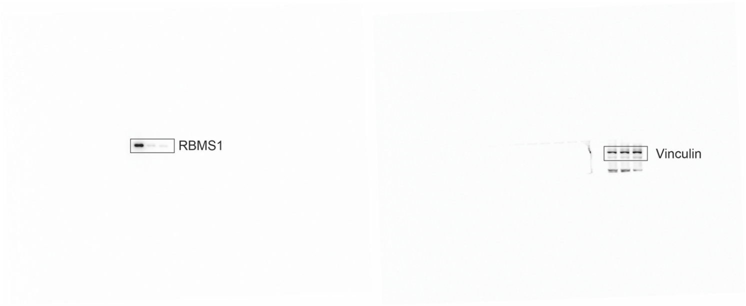

Supplementary Fig.5E(HEK-293T)

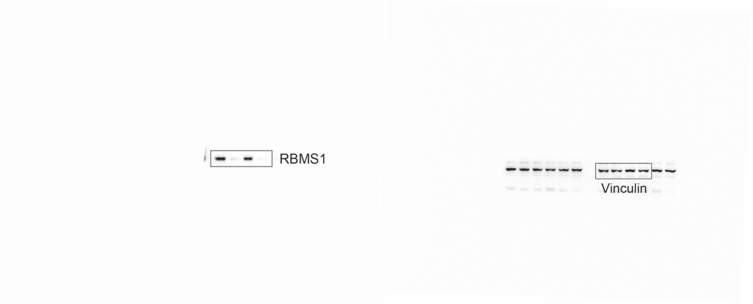

Supplement: Supplementary file 10 — Supplemental Figure S7 Original uncut gel figures [file 41418_2022_1012_MOESM10_ESM.pdf]
