# Supplementary material for: Loss of RBMS1 promotes anti-tumor immunity through enabling PD-L1 checkpoint blockade in triple-negative breast cancer
Source: Cell Death Differ. 2022 May 10;29(11):2247–61. doi: 10.1038/s41418-022-01012-0 (PMC9613699; doi:10.1038/s41418-022-01012-0)
Supplement: Supplementary file 11 — signed Author contribution form [file 41418_2022_1012_MOESM11_ESM.pdf]

DECLARATION OF CONTRIBUTIONS TO ARTICLE

ADMC

Manuscript Number: CDD-21-2791

Journal Name: *Cell Death & Differentiation* (the 'Journal')

Proposed Title of the Contribution:

Loss of RBMS1 promotes anti-tumor immunity through enabling PD-L1 checkpoint blockade in triple-negative breast cancer (the 'Contribution)

Author(s):

Jinrui Zhang, Ge Zhang, Wenjing Zhang, Lu Bai, Luning Wang, Tiantian Li, Li Yan, Yang Xu, Dan Chen, Wenting Gao, Chuanzhou Gao, Chaoqun Chen, Menglin Ren, Yuexia Jiao, Hongqiang Qin, Yu Sun, Lili Zhi, Yangfan Qi, Jinyao Zhao, Quentin Liu, Han Liu, Yang Wang (the 'Authors')

For all *CDD* articles, each person named as an author in the published version must be able to show he or she has contributed substantially to the article.

Authorship credit should be based on 1) substantial contributions to conception and design, acquisition of data, or analysis and interpretation of data; 2) drafting the article or revising it critically for important intellectual content; and 3) final approval the version to be published. Authors should meet conditions 1, 2 and 3.

Any person who cannot be shown to have made a substantial contribution to the article cannot be listed as an author in the final version. The name of any person who is deemed to have made a minor contribution can, however, appear in the Acknowledgments section of the article.

Please complete the table below to indicate the contributions of all named authors to the manuscript.

Please complete the table below to indicate the contributions of all named authors to the figures.

Author Full Name	Specification of Contribution to the Manuscript:
Yang Wang	Conception and design and wrote the manuscript.
Jinrui Zhang, Ge Zhang	Acquisition of data and analyzed data, wrote and reviewed the manuscript.
Wenjing Zhang, Lu Bai	Acquisition of data and analyzed data, wrote and reviewed the manuscript.
Luning Wang, Tiantian Li	Collected data and analyzed data.
Li Yan, Yang Xu	Performed CAR-T experiment.
Dan Chen, Wenting Gao, Chuanzhou Gao	Performed and/or provided help with the IHC assay.
Menglin Ren, Yuexia Jiao	Collected data.
Hongqiang Qin and Chaoqun Chen	IP-LC/MS assay and Bioinformatics Analysis.
Yu Sun, Lili Zhi	Performed and/or provided help with the PLA assay.
Yangfan Qi, Jinyao Zhao	Collected data.
Quentin Liu, Han Liu	Data analysis and advised about the manuscript.
All authors	All authors discussed the results and commented on the manuscript.

Figure 1:

JZ, GZ, LB and CC collected the data and assembled the figure, JZ and DC analyzed the data.

Figure 2:

JZ and GZ and DC and TL collected the data, JZ analyzed the data.

Figure 3:

WZ and LW collected the data and assembled the figure, WZ analyzed the data.

Figure 4：

WZ and LB and LZ collected the data and assembled the figure.

Figure 5:

WZ and YS and YJ collected the data and assembled the figure, LW analyzed the data.

Figure 6:

JZ and GZ and WG collected the data and assembled the figure, JZ analyzed the data.

Figure 7:

JZ and LY and YX and CG collected the data and assembled the figure, GZ and JZ analyzed the data.

Figure 8:

CC and GZ assembled the figure， JZ analyzed the data.

Signed for and on behalf of the Author(s):	Print Name:	Date:
	Yang Wang	22. Feb. 2022
