# Supplementary material for: Loss of RBMS1 promotes anti-tumor immunity through enabling PD-L1 checkpoint blockade in triple-negative breast cancer
Source: Cell Death Differ. 2022 May 10;29(11):2247–61. doi: 10.1038/s41418-022-01012-0 (PMC9613699; doi:10.1038/s41418-022-01012-0)
Supplement: Supplementary file 13 — signed change of authorship request form [file 41418_2022_1012_MOESM13_ESM.pdf]

SPRINGER NATURE

Change of authorship request form - Journals (pre-acceptance)

Section 1: Please provide the current title of manuscript

Manuscript ID no.: CDD-21-2791

Title: Loss of RBMS1 promotes anti-tumor immunity through enabling PD-L1 checkpoint blockade in triple-negative breast cancer

Section 2: Please provide the previous authorship, in the order shown on the manuscript before the changes were introduced. Please indicate the corresponding author by adding (CA) behind the name.

	First name(s)	Family name	ORCID or SCOPUS id, if available
1st author	Jinrui	Zhang	
2nd author	Ge	Zhang	
3rd author	Wenjing	Zhang	
4th author	Lu	Bai	
5th author	Luning	Wang	
6th author	Tiantian	Li	
7th author	Li	Yan	
8th author	Chaoqun	Chen	
9th author	Menglin	Ren	
10th author	Yuexia	Jiao	

Please use an additional sheet if there are more than 10 authors.

SPRINGER NATURE Springer Nature is one of the world's leading global research, educational and professional publishers, created in May 2015 through the combination of Nature Publishing Group, Palgrave Macmillan, Macmillan Education and Springer Science+Business Media.

11th author	Yu	Sun	
12th author	Lili	Zhi	
13th author	Yangfan	Qi	
14th author	Jinyao	Zhao	
15th author	Yang	Wang (CA)	0000-0001-9385-7393

SPRINGER NATURE

Change of authorship request form - Journals (pre-acceptance)

Section 3: Please provide a justification for change. Please use this section to explain your reasons for changing the authorship of your manuscript, e.g. what necessitated the change in authorship? Please refer to the (journal) policy pages for more information about authorship. Please explain why omitted authors were not originally included and/or why authors were removed on the submitted manuscript.

In the revision of this manuscript, several students and colleagues helped to perform new required experiments and interpret the newly obtained data. Based on their contributions, they are included as new contributing authors in the revised manuscript.

Section 4: Proposed new authorship. Please provide your new authorship list in the order you would like it to appear on the manuscript. Please indicate the corresponding author by adding (CA) behind the name. If the Corresponding Author has changed, please indicate the reason under section 3.

	First name(s)	Family name (this name will appear in full on the final publication and will be searchable in various abstract and indexing databases)	Affiliated institute	E-mail address
1st author	Jinrui	Zhang	Dalian Medical University	1173432719@qq.com
2nd author	Ge	Zhang	Dalian Medical University	gezhangunc@163.com
3rd author	Wenjing	Zhang	Dalian Medical University	wenjing.1900@aliyun.com
4th author	Lu	Bai	Dalian Medical University	ellebai@foxmail.com
5th author	Luning	Wang	Dalian Medical University	LNWang0620@163.com
6th author	Tiantian	Li	Dalian Medical University	lingyvxuan@163.com
7th author	Li	Yan	Dalian Medical University	18379798168@126.com
8th author	Yang	Xu	Southern University of Science and Technology	xuy6@sustech.edu.cn
9th author	Dan	Chen	Dalian Medical University	chendan_youxiang@163.com
10th author	Wenting	Gao	Dalian Medical University	gwtpkwy@163.com

Please use an additional sheet if there are more than 10 authors.

SPRINGER NATURE Springer Nature is one of the world's leading global research, educational and professional publishers, created in May 2015 through the combination of Nature Publishing Group, Palgrave Macmillan, Macmillan Education and Springer Science+Business Media.

11th author	Chuanzhou	Gao	Dalian Medical University	gaocz-2008@163.com
12th author	Chaoqun	Chen	Dalian Medical University	veryqun@icloud.com
13th author	Menglin	Ren	Dalian Medical University	a824116@163.com
14th author	Yuexia	Jiao	Dalian Medical University	1926630694@qq.com
15th author	Hongqiang	Qin	Dalian Institute of Chemical Physics, Chinese Academy of Sciences,	qinhq@dicp.ac.cn
16th author	Yu	Sun	Dalian Medical University	18754809573@163.com
17th author	Lili	Zhi	Dalian Medical University	zhi931227@163.com
18th author	Yangfan	Qi	Dalian Medical University	yangfanshy@163.com
19th author	Jinyao	Zhao	Dalian Medical University	jinyao_z@163.com
20th author	Quentin	Liu	Dalian Medical University	liuq9@mail.sysu.edu.cn
21st author	Han	Liu	Dalian Medical University	liuhan@dmu.edu.cn
22nd author	Yang	Wang(CA)	Dalian Medical University	yangwang@dmu.edu.cn

SPRINGER NATURE

Change of authorship request form - Journals (pre-acceptance)

Section 5: Author contribution, Acknowledgement and Disclosures. Please use this section to provide a new disclosure statement and, if appropriate, acknowledge any contributors who have been removed as authors and ensure you state what contribution any new authors made (if applicable per the journal or book (series) policy). Please ensure these are updated in your manuscript - <u>after approval of the change(s)</u> - as our production department will not transfer the information in this form to your manuscript.

New acknowledgements:

Not applicable

New Disclosures (financial and non-financial interests, funding):

The authors have declared that no conflict of interest exists.

New Author Contributions statement (if applicable per the journal policy):

Y.W. conceived the project and designed the experiments. J. Z., G. Z., W. Z., L. B., L. W., T. L., L. Y., M. R., Y. J., H. Q., Y. S., L. Z., Y. Q., and J. Z. designed and performed most of the experiments, whereas Y. X., D. C., W. G., C. G., C. C., Q. L., and H. L. performed data analysis. Y. W. provided funds. Y. W. wrote the manuscript.

State 'Not applicable' if there are no new authors.

SPRINGER NATURE Springer Nature is one of the world's leading global research, educational and professional publishers, created in May 2015 through the combination of Nature Publishing Group, Palgrave Macmillan, Macmillan Education and Springer Science+Business Media.

SPRINGER NATURE Change of authorship request form - Journals (pre-acceptance)

Section 6: Declaration of agreement. *All* authors, unchanged, new and removed *must* sign this declaration.
(NB: Please print the form, (docu)-sign and return/upload a scanned copy. Please note that signatures that have been inserted as an image file are acceptable as long as it is handwritten. Typed names in the signature box are unacceptable.) *** Please delete as appropriate. Delete all of the bold if you were on the original authorship list and are remaining as an author.**

	First name	Family name		Signature	Date
1st author	Jinrui	Zhang	I agree to the proposed new authorship shown in section 4 /~~**and the addition/removal*of my name to the authorship list**~~ /and the proposed change in corresponding author	Jin Rui Zhang	02/20/2022
2nd author	Ge	Zhang	I agree to the proposed new authorship shown in section 4 /~~**and the addition/removal*of my name to the authorship list**~~ /and the proposed change in corresponding author	GZhang	02/20/2022
3rd author	Wenjing	Zhang	I agree to the proposed new authorship shown in section 4 /~~**and the addition/removal*of my name to the authorship list**~~ /and the proposed change in corresponding author	Wenjing Zhang	02/20/2022
4th authors	Lu	Bai	I agree to the proposed new authorship shown in section 4 /~~**and the addition/removal*of my name to the authorship list**~~ /and the proposed change in corresponding author	Lu Bai	02/20/2022
5th author	Luning	Wang	I agree to the proposed new authorship shown in section 4 /~~**and the addition/removal*of my name to the authorship list**~~ /and the proposed change in corresponding author	Luning Wang	02/20/2022
6th author	Tiantian	Li	I agree to the proposed new authorship shown in section 4 /~~**and the addition/removal*of my name to the authorship list**~~ /and the proposed change in corresponding author	Tiantian Li	02/20/2022
7th author	Li	Yan	I agree to the proposed new authorship shown in section 4 /~~**and the addition/removal*of my name to the authorship list**~~ /and the proposed change in corresponding author	Li Yan	02/20/2022

SPRINGER NATURE Change of authorship request form - Journals (pre-acceptance)

	First name	Family name		Signature	Date
8th author	Yang	Xu	I agree to the proposed new authorship shown in section 4 /**and the addition/~~removal*~~of my name to the authorship list** /and the proposed change in corresponding author	Yang Xu	02/20/2022
9th author	Dan	Chen	I agree to the proposed new authorship shown in section 4 /**and the addition/~~removal*~~of my name to the authorship list** /and the proposed change in corresponding author	Dan Chen	02/20/2022
10th author	Wenting	Gao	I agree to the proposed new authorship shown in section 4 /**and the addition/~~removal~~*of my name to the authorship list** /and the proposed change in corresponding author	Wenting Gao	02/20/2022

Please use an additional sheet if there are more than 10 authors.

In case of author collaborations with formal agreement:

	Name of consortium/consortia	First name	Family name		Signature	Date
Representative/legal guarantor				I agree to the proposed new authorship shown in section 4 /**and the addition/removal*of my name to the authorship list** /and the proposed change in corresponding author		

Both added/removed authors should complete the information in the first table under Section 6.

---- End of form ----

SPRINGER NATURE Springer Nature is one of the world's leading global research, educational and professional publishers, created in May 2015 through the combination of Nature Publishing Group, Palgrave Macmillan, Macmillan Education and Springer Science+Business Media.

11th author	Chuanzhou	Gao	I agree to the proposed new authorship shown in section 4 /**and the addition~~/removal~~*of my name to the authorship list** /and the proposed change in corresponding author	Chuanzhou Gao	02/20/2022
12th author	Chaoqun	Chen	I agree to the proposed new authorship shown in section 4 /~~**and the addition/removal*of my name to the authorship list**~~ /and the proposed change in corresponding author	Chaoqun chen	02/20/2022
13th author	Menglin	Ren	I agree to the proposed new authorship shown in section 4 /~~**and the addition/removal*of my name to the authorship list**~~ /and the proposed change in corresponding author	Menglin Ren	02/20/2022
14th author	Yuexia	Jiao	I agree to the proposed new authorship shown in section 4 /~~**and the addition/removal*of my name to the authorship list**~~ /and the proposed change in corresponding author	Yuexia Jiao	02/20/2022
15th author	Hongqiang	Qin	I agree to the proposed new authorship shown in section 4 /**and the addition~~/removal~~*of my name to the authorship list** /and the proposed change in corresponding author	Hongqiang Qin	02/20/2022
16th author	Yu	Sun	I agree to the proposed new authorship shown in section 4 /~~**and the addition/removal*of my name to the authorship list**~~ /and the proposed change in corresponding author	Yu Sun	02/20/2022
17th author	Lili	Zhi	I agree to the proposed new authorship shown in section 4 /~~**and the addition/removal*of my name to the authorship list**~~ /and the proposed change in corresponding author	LiLi Zhi	02/20/2022
18th author	Yangfan	Qi	I agree to the proposed new authorship shown in section 4 /~~**and the addition/removal*of my name to the authorship list**~~ /and the proposed change in corresponding author	Yangfan Qi	02/20/2022
19th author	Jinyao	Zhao	I agree to the proposed new authorship shown in section 4 /~~**and the addition/removal*of my name to the authorship list**~~ /and the proposed change in corresponding author	Jinyao Zhao	02/20/2022
20th author	Quentin	Liu	I agree to the proposed new authorship shown in section 4 /**and the addition~~/removal~~*of my name to the authorship list** /and the proposed change in corresponding author	[illegible]	02/20/2022
21st author	Han	Liu	I agree to the proposed new authorship shown in section 4 /**and the addition~~/removal~~*of my name to the authorship list** /and the proposed change in corresponding author	[illegible]	02/20/2022
22nd author	Yang	Wang	I agree to the proposed new authorship shown in section 4 /~~**and the addition/removal*of my name to the authorship list**~~ /and the proposed change in corresponding author	[illegible]	02/20/2022
